# Heterotrimeric G-protein subunits regulate plant architecture, pod development, seed size, and symbiotic nodulation in* Medicago truncatula*

**DOI:** 10.1007/s42994-025-00210-x

**Published:** 2025-05-07

**Authors:** Fanghao Sun, Fugui Zhu, Shasha Ran, Qinyi Ye, Tao Wang, Jiangli Dong

**Affiliations:** 1 College of Biological Sciences, China Agricultural University, Beijing, 100193 China; 2 Key Laboratory of Forage and Endemic Crop Biology, Ministry of Education, College of Life Sciences, Inner Mongolia University, Hohhot, 010000 China

**Keywords:** G proteins, Regulator of G-protein signaling, *Medicago truncatula*, CRISPR/Cas9, Symbiotic nodulation

## Abstract

Heterotrimeric G proteins are crucial transducers of signaling from receptors, participating in growth and development, as well as in responses to biotic and abiotic stimuli. However, little is known about their roles in regulating various yield-related traits in legumes. In this study, we systematically analyzed the functions of two G-protein-encoding genes, *MtGα1* and *MtGβ1*, along with *Regulator of G-protein Signaling1* (*MtRGS1*), in *Medicago truncatula*. All three genes were ubiquitously expressed in roots, stems, leaves, nodules, flowers, and pods. We generated the knockout mutants *Mtgα1*, *Mtgβ1*, and *Mtrgs1* using CRISPR/Cas9 and assessed their growth and development. *MtGα1* knockout resulted in slightly shorter plants with smaller pods and shorter spines, but larger seeds, without affecting overall biomass or other traits. *MtGβ1* knockout led to dwarfism, weak root development, a severe drop in biomass production, smaller legume pods with shorter spines, and smaller seeds. However, the *Mtrgs1* mutants were largely similar to wild-type plants, with few significant defects in growth and development. We also investigated the symbiotic nodulation-related phenotypes of these mutants, discovering that *Mtgβ1* mutants produce lighter nodules, whereas *Mtgα1* and *Mtrgs1* mutants have normal nodulation phenotypes similar to those of wild-type plants. These observations suggest that MtGβ1 positively regulates nodulation, although the detailed mechanisms by which G proteins regulate symbiotic nitrogen fixation in *M. truncatula* remain to be explored. This work provides potentially valuable genetic resources for further functional analysis and elucidation of the molecular mechanisms of G proteins in this model legume.

**Supplementary Information:**

The online version contains supplementary material available at 10.1007/s42994-025-00210-x.

## Introduction

Heterotrimeric GTP-binding proteins (G proteins), consisting of Gα, Gβ, and Gγ subunits, transmit extracellular signals to downstream effectors in all eukaryotes. The GDP-bound form of Gα associates with the Gβγ dimer to form an inactive heterotrimer during the resting phase. Upon activation, the GDP within Gα is exchanged for GTP, leading to the release of the Gβγ dimer from Gα. Both GTP-bound Gα and the Gβγ dimer can then interact with various downstream components to transduce signals (Gilman 1987). The GTPase activity of the Gα subunit catalyzes the hydrolysis of its bound GTP to GDP, resulting in the reassociation of GDP-bound Gα with the Gβγ dimer to reconstitute the inactive heterotrimer, which is then ready to be reactivated (Urano et al. 2013). The intrinsic rate of GTP hydrolysis by Gα is relatively slower than the rate of GDP-to-GTP exchange, and this hydrolysis is accelerated by GTPase-activating or GTPase-accelerating proteins (GAPs), such as regulators of G-protein signaling (RGS) proteins (McCudden et al. 2005). Guanine nucleotide disassociation inhibitors (GDIs) inhibit the rate of GDP release from the Gα subunit, whereas guanine nucleotide exchange factors (GEFs), such as G-protein-coupled receptors (GPCRs), modulate the rate of GDP-to-GTP exchange. Collectively, GDIs, GEFs, and GAPs are crucial for the fine-tuning of signal transduction events (Mohanasundaram and Pandey 2023).

Compared to metazoans, plants possess relatively few canonical G proteins (Jones and Assmann 2004). For instance, the genomes of Arabidopsis (*Arabidopsis thaliana*), rice (*Oryza sativa*), and maize (*Zea mays*) each encode only one canonical Gα subunit and one Gβ subunit, whereas the larger, paleopolyploid genome of soybean (*Glycine max*) encodes four canonical Gα subunits and four Gβ subunits; for comparison, the human genome encodes 23 distinct Gα subunits and five Gβ subunits (Gao et al. 2019; Offermanns 2003; Ullah et al. 2003; Wettschureck and Offermanns 2005; Wu et al. 2020). Notably, another type of Gα proteins, the extra-large G proteins (XLGs), occur specifically in plants. XLGs have more subfamily members and are usually larger than canonical Gα proteins, as their name suggests. XLGs can also form G-protein trimers, but they lack key residues for GTP binding (Maruta et al. 2021a, b; Sharma et al. 2023). RGSs are not highly conserved compared to the core G proteins components in plants. Arabidopsis and soybean have one and two RGS proteins, respectively, whereas *RGS* homologs have been lost from the genomes of rice, maize, and many other monocot species (Chen et al. 2003; Choudhury et al. 2012). Another G-protein component, the Gγ subunit, expands the diversity of plant G-protein networks, as these subunits are encoded by several genes, such as Arabidopsis encodes three Gγ subunits and rice encodes five Gγ subunits (Thung et al. 2012; Xu et al. 2019). They interact with the Gβ subunit to form dimers, thereby modulating a variety of responses (Dong et al. 2023; Urano et al. 2016).

Phenotypic analyses in Arabidopsis, rice, maize, and other plant species have revealed that G proteins are key regulators of plant growth and development. An Arabidopsis mutant defective in Gα, *gpa1*, has hypocotyls, flowers, siliques, and seeds that are shorter and wider, to varying degrees, than those in the wild type, together with lower root biomass (Chen et al. 2006a; Ullah et al. 2003). Mutation of *Gα* in rice and maize leads to severe impairment of plant growth, including decreases in plant height, root growth, and yield (Bommert et al. 2013; Izawa et al. 2010; Oki et al. 2009; Pathak et al. 2021; Urano et al. 2015). The Arabidopsis null mutant of *Gβ*, *agb1*, displays a more pronounced shortening of hypocotyls, leaves, petioles, flowers, siliques and seeds than the *gpa1* mutant, along with an increase in root biomass (Chen et al. 2006a; Ullah et al. 2003). Knockout of the *Gβ* gene in monocot species such as rice and maize, and in some dicot species such as tomato (*Solanum lycopersicum*), results in seedling death, in contrast to the Arabidopsis *agb1* mutant, which is viable. Therefore, mechanistic studies of Gβ have been conducted primarily in Arabidopsis (Gao et al. 2019; Ninh et al. 2021; Utsunomiya et al. 2011; Wu et al. 2020). Plants that overexpress an *RGS* or have suppressed *Gα* expression display similar phenotypes. For example, overexpression of *RGS1* in Arabidopsis resulted in shorter plants with shorter petioles, akin to the phenotypes observed in the *gpa1* mutant (Chen et al. 2006b). Given that many plants lack RGS proteins, research on RGS has also predominantly focused on Arabidopsis. In addition to their developmental roles, G proteins occupy significant positions in the interplay between plants and microbes. Several studies have demonstrate that Gα, Gβ, and RGS are intricately involved in the signaling pathway activated by the pathogen effector flg22 in Arabidopsis and in nodulation in soybean (Choudhury and Pandey 2013; Liang et al. 2016; Xue et al. 2020).

Legumes are highly valued for their nutritional value and their ability to engage in symbiotic nitrogen fixation, and thus play an important role in food security and sustainable agriculture (Beltrán and Cañas 2018). Organ and tissue development, metabolism, and stress responses in legumes can show marked differences from those of the well-studied model plant Arabidopsis (Cañas and Beltrán 2018). Research into G-protein signaling in legumes has concentrated on the regulatory mechanisms of the legume–rhizobium symbiosis that is central to the formation of nodules and nitrogen fixation. During nodule formation in soybean, Nod factor receptor 1 (NFR1) interacts with RGS and Gα proteins, with the phosphorylation of RGS by NFR1 maintaining Gα in an inactive conformation (Choudhury and Pandey 2013, 2015). At the same time, Symbiosis receptor kinase (SymRK), an integral component of the nodule receptor complex, interacts with and phosphorylates Gα and RGS. Phosphorylated RGS exhibits enhanced GTPase-accelerating activity, further promoting the conversion of Gα from the active to the inactive form and thereby allowing constitutive signaling from the freed Gβγ dimer (Choudhury and Pandey 2022, 2024). A study using the hairy root transformation technique demonstrated that the RNA interference (RNAi)-based knockdown of *Gβ* transcript levels in the A17 genotype of *Medicago truncatula* or pea (*Pisum sativum*), both of which form indeterminate nodules, resulted in the formation of fewer nodules. This suggest that Gβ has a positive effect on nodule formation in these two legumes, aligning with observations in soybean, which forms determinate nodules (Bovin et al. 2022; Choudhury and Pandey 2013). Determinate nodules differ from indeterminate nodules by lacking the persistent nodule meristem (Kohlen et al. 2018). Whether Gα and RGS have analogous functions in other legumes to those in soybean requires investigation. In addition, few studies have looked into other aspects of G proteins in legumes, especially in plant development and agronomic traits.

*Medicago truncatula* is an ideal model legume for investigating plant development and indeterminate nodulation, offering significant advantages for research into analogous traits in leguminous crops, especially its close relative, alfalfa (*Medicago sativa*). Reports of efficient gene editing via clustered regularly interspaced short palindromic repeats (CRISPR)/CRISPR-associated nuclease 9 (Cas9) technology has accelerated reverse genetics research in *M. truncatula* (Meng et al. 2017; Wolabu et al. 2020; Zhu et al. 2021). In this study, we characterize the components of heterotrimeric G proteins and the RGS of *M. truncatula*. We generated the first set of stably edited mutants in G-protein genes in legumes and investigated the developmental and reproductive phenotypes of the resulting *Mtgα1*, *Mtgβ1*, and *Mtrgs1* mutants, expanding our understanding of the functions of G proteins in forage legumes. The roles of G proteins in development and symbiotic nitrogen fixation in *M. truncatula* showed both similarities and differences compared to those in other crops, supporting the idea that G-protein signaling networks vary across different lineages.

## Results

### Analysis of G-protein components in *Medicago truncatula*

To identify the genes encoding individual G-protein subunits and RGS in *M. truncatula*, we conducted a BLAST search, using the predicted amino acid sequences for Gα, Gβ, Gγ, and RGS proteins from Arabidopsis and rice as queries. We then reconstructed the corresponding phylogenetic trees, using the protein sequences for all related proteins from *M. truncatula*, *Lotus japonicus*, soybean, black-eyed pea (*Vigna unguiculata*), Arabidopsis, tomato, rice, maize, and sorghum (*Sorghum bicolor*). Previous studies have indicated that the *M. truncatula* genome harbors two *Gα* genes, five *XLG* genes (Fig. S1), one *Gβ* gene (Fig. S2), and five *Gγ* genes (Fig. S3) (Bovin et al. 2022). In this study, we discovered two additional *Gγ* genes (MtrunA17_Chr1g0188131, Type I Gγ; MtrunA17_Chr4g0070381, Type III Gγ) (Fig. S3) and one more *RGS* gene (MtrunA17_Chr3g0123191) (Fig. S4) in the *M. truncatula* genome (Mt5.0), as confirmed by their positions in the phylogenetic trees. We conclude that the Gα, Gβ, and RGS proteins are encoded in *M. truncatula* by only one or two genes, suggesting that they might play critical roles in various aspects of plant life.

**Fig. 1:**
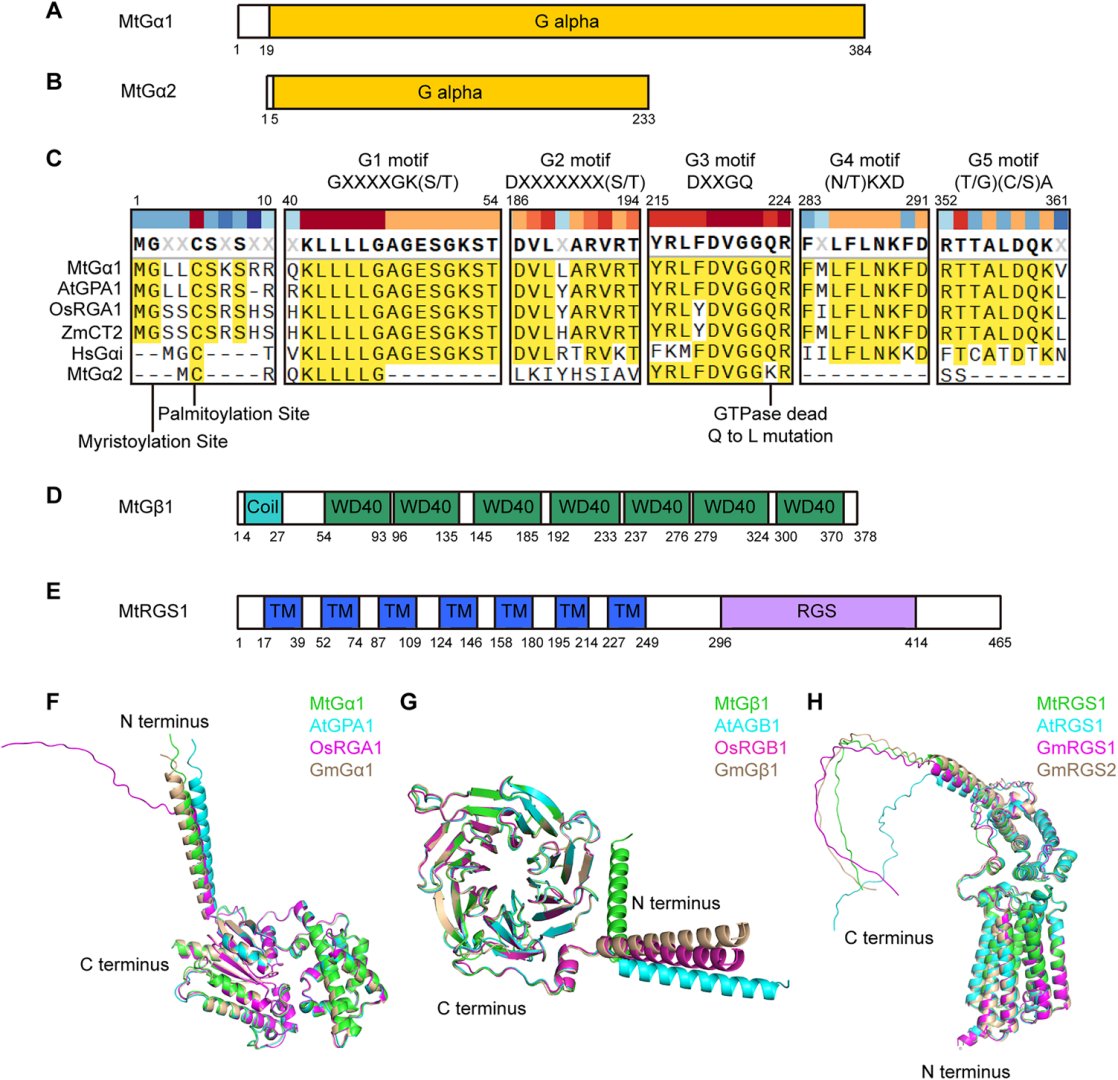
Predicted structures of Gα, Gβ, and RGS from *M. truncatula.* **A**, **B** Diagrams of MtGα1 (MtrunA17_Chr1g0151141) (**A**) and MtGα2 (MtrunA17_Chr3g0136391) (**B**). The domains were predicted using the SMART website (https://smart.embl.de/). Numbers represents the amino acid positions. **C** Multiple sequence alignment of the N-terminal modification sites and conserved G1–G5 motifs of Gα proteins. MtGα1 and MtGα2, *Medicago truncatula*; AtGPA1, *Arabidopsis thaliana*; OsRGA1, *Oryza sativa*; ZmCT2, *Zea mays*; HsGαi, *Homo sapiens*. **D**, **E** Diagrams of MtGβ1 (MtrunA17_Chr3g0144511) (**D**) and MtRGS1 (MtrunA17_Chr3g0123191) (**E**). The domains were predicted using the SMART website. Numbers represents the amino acid positions. TM, transmembrane domain. **F**–**H** Comparative studies with the AlphaFold3 models of Gα (**F**), Gβ (**G**), and RGS (**H**) proteins. **F** Superimposition of the predicted structures of MtGα1 (green), AtGPA1 (cyan), OsRGA1 (magenta), and GmGα1 (wheat). **G** Superimposition of the predicted structures of MtGβ1 (green), AtAGB1 (cyan), OsRGB1 (magenta), and GmGβ1 (wheat). **H** Superimposition of the predicted structures of MtRGS1 (green), AtRGS1 (cyan), GmRGS1 (magenta), and GmRGS2 (wheat)

Protein domain predictions using the SMART website (https://smart.embl.de/) revealed that MtGα1 and MtGα2 each have a Gα domain, but the Gα domain of MtGα2 is shorter than that of MtGα1 (Fig. 1A, B). The phylogenetic tree indicates that MtGα2 is genetically distant from other Gα proteins (Fig. S1). MtGα1 contains the conserved G1–G5 motifs typical of canonical Gα proteins, as well as an N-terminal myristoylation site and a palmitoylation site (Fig. 1A, C). By contrast, MtGα2 only harbors the G3 motif and the palmitoylation site (Fig. 1B, C). In addition, the key site for GTPase activity within the G3 motif of MtGα2 is a lysine (K) residue instead of the conserved glutamine (Q) residue present in other Gαs (Fig. 1C). This suggests that MtGα2 may not be a functional Gα protein. The subunit MtGβ1 contains N-terminal coiled-coil helices and seven WD40 domains (Fig. 1D), whereas MtRGS1 contains seven transmembrane domains in its N-terminal half and a cytoplasmic RGS domain in its C-terminal half (Fig. 1E), which are consistent with the structures of canonical Gβs and RGSs in plants (Urano et al. 2013). We used the 3D models predicted by AlphaFold for MtGα1, MtGα2, MtGβ1, and MtRGS1 to explore their protein structures (Fig. 1F–H, Fig. S5). The predicted models of MtGα1, MtGβ1, and MtRGS1 showed structures that are conserved with respect to those of the Gα, Gβ, and RGS proteins in Arabidopsis, rice, and soybean, respectively (Fig. 1F–H). However, the predicted 3D structure of MtGα2 appeared to lack the helix normally present in the N terminus of Gα subunits (Fig. S5). Therefore, we focused on *MtGα1*, *MtGβ1*, and *MtRGS1* in this study.

### Expression patterns of *MtGα1*, *MtGβ1*, and *MtRGS1*

**Fig. 2:**
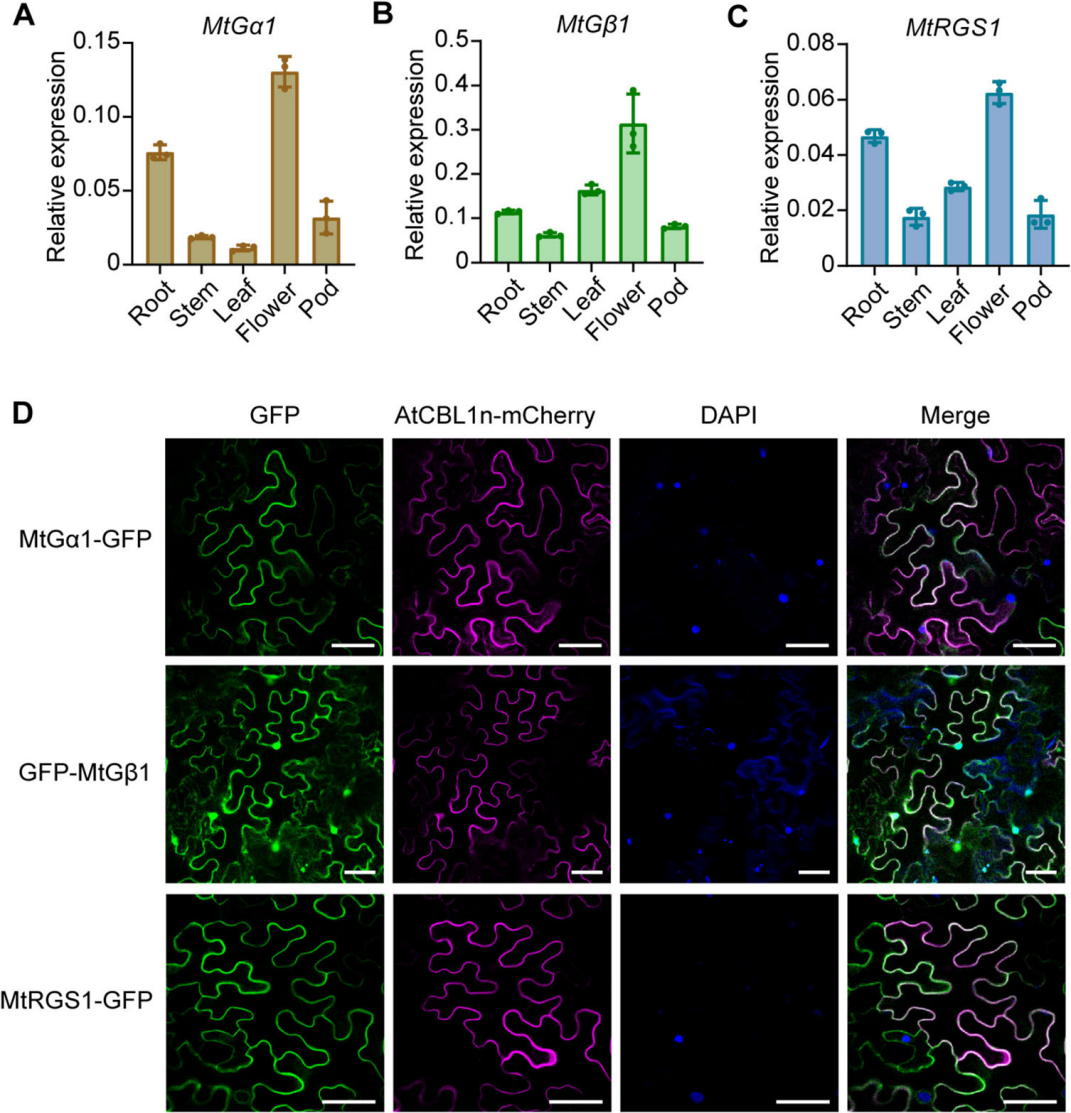
Expression patterns of *MtGα1*, *MtGβ1* and *MtRGS1* and subcellular localization of their encoding proteins*.* **A**–**C** RT-qPCR analysis of *MtGα1* (**A**), *MtGβ1* (**B**), and *MtRGS1* (**C**) expression levels in the root, stem, leaf, flower, and pod tissues of R108 under normal growth conditions. The relative expression level of each gene was normalized to that of *MtACTIN4A*. Values are means ± standard deviation (SD) from three biological replicates. **D** Subcellular localization of MtGα1-GFP, GFP-MtGβ1, and MtRGS1-GFP in *N. benthamiana* leaves. *AtCBL1n-mCherry* was co-expressed as a plasma membrane marker. Nuclei were stained with DAPI. Scale bars, 50 μm

To investigate the expression patterns of genes encoding G-protein subunits in *M. truncatula*, we analyzed the transcript levels of *MtGα1*, *MtGβ1*, and *MtRGS1* across different tissues in the wild-type R108 by RT-qPCR. *MtGα1*, *MtGβ1*, and *MtRGS1* were all expressed in roots, stems, leaves, flowers, and pods, with *MtGβ1* being the most highly expressed (Fig. 2A–C).

For subcellular localization analysis, considering the N-terminal modification sites of MtGα1 (Fig. 1C) and the N-terminal seven transmembrane domains of MtRGS1 (Fig. 1E), we generated constructs with the full-length coding sequence of *MtGα1* or *MtRGS1* placed in-frame and upstream of the sequence for the green fluorescent protein (*GFP*); for MtGβ1, we generated two constructs, encoding fusions of MtGβ1 with GFP to its C terminus or N terminus, respectively. We then introduced each plasmid along with *35S:AtCBL1n*-*mCherry* (a plasma membrane localization marker encoding Arabidopsis CALCINEURIN B-LIKE PROTEIN fused to the red fluorescent protein mCherry) into the leaves of *Nicotiana benthamiana* plants via Agrobacterium (*Agrobacterium tumefaciens*)-mediated infiltration. We detected green fluorescence showed at the plasma membrane for MtGα1-GFP and MtRGS1-GFP (Fig. 2D). We did not observe visible fluorescence for MtGβ1-GFP, possibly due to low abundance. By contrast, we observed green fluorescence for GFP-MtGβ1 in the nucleus, cytoplasm, and plasma membrane (Fig. 2D). These results indicate that the localization patterns of MtGα1, MtGβ1, and MtRGS1 are consistent with those of canonical G proteins in other plant species (Anderson and Botella 2007; Bisht et al. 2011; Chen et al. 2003; Choudhury et al. 2012).

### Construction of mutants of *MtGα1*, *MtGβ1* and *MtRGS1* by CRISPR/Cas9

**Fig. 3:**
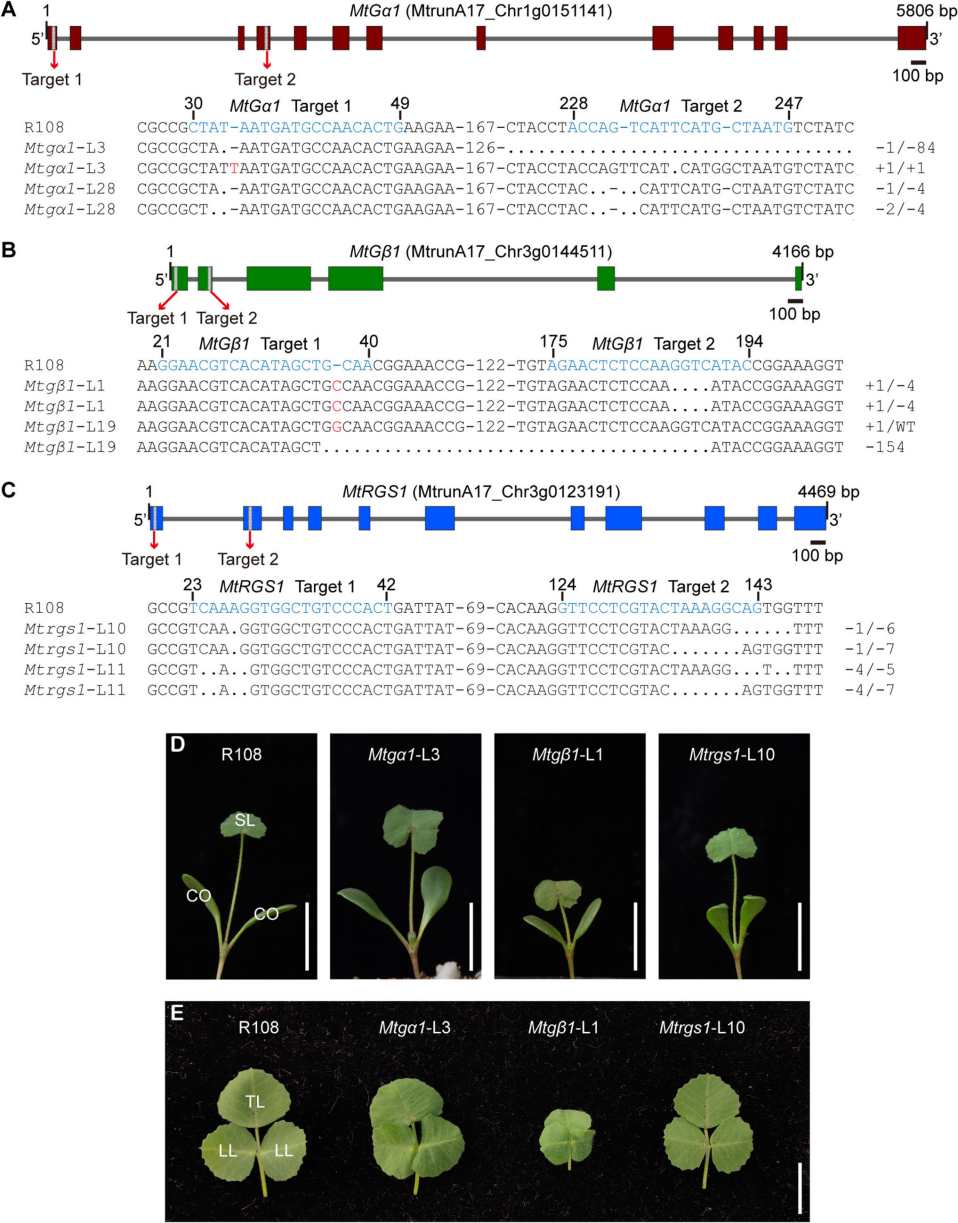
Characterization of gene-edited *Mtgα1*, *Mtgβ1*, and *Mtrgs1* mutants in *M. truncatula.* **A**–**C** Diagrams showing the gene models and single guide RNA (sgRNA) target sites for CRISPR/Cas9-mediated editing of *MtGα1* (**A**), *MtGβ1* (**B**), and *MtRGS1* (**C**) and the sequences of the mutants at the target site. In the gene models, exons are denoted by boxes. Introns are depicted as gray lines, and the positions of the sgRNA target sites are shown as gray boxes. In the sequence alignments, the target sites are indicated in blue. Nucleotides in red represent insertions, and dots represent deletions. **D** Representative photographs of R108, *Mtgα1*, *Mtgβ1*, and *Mtrgs1* seedlings at 7 days post-germination. Scale bars, 5 cm. CO, cotyledon; SL, single leaf. **E** Representative photograph of the third compound leaves from R108, *Mtgα1*, *Mtgβ1*, and *Mtrgs1* seedlings at 4 weeks post-germination. Scale bars, 1 cm. TL, terminal leaflet; LL, lateral leaflet

To dissect the biological functions of MtGα1, MtGβ1, and MtRGS1 *in planta*, we applied a *Medicago*-optimized CRISPR/Cas9 toolkit (Zhu et al. 2021) to individually target *MtGα1*, *MtGβ1*, and *MtRGS1* in the R108 background. We chose two target sites per gene, with the goal of introducing mutations at the beginning of the coding region, thereby raising our chances of obtaining mutants with a nonfunctional protein (Fig. 3A–C). We obtained multiple T0 transgenic plants in R108 by *Agrobacterium*-mediated transformation, after which we determined the presence and type of mutations present in each positive transgenic plant by PCR amplification of the targeted genomic region. We successfully identified two distinct mutant plants for each gene by Sanger sequencing analysis. *Mtgα1*-L3 is a biallelic mutant, featuring a 1-bp deletion (at target site 1) and an 84-bp deletion (at target site 2) in one allele and 1-bp insertions at both target sites in the other allele; *Mtgα1*-L28 also carries biallelic mutations, with a 1-bp deletion (target site 1) and a 4-bp deletion (target site 2) in one allele and a 2-bp deletion (target site 1) and a 4-bp deletion (target site 2) in the other allele (Fig. 3A, Fig. S6). *Mtgβ1*-L1 harbors homozygous mutations with a 1-bp insertion (target site 1) and a 4-bp deletion (target site 2), whereas *Mtgβ1*-L19 is biallelic with a 1-bp insertion (target site 1) in one allele and a 1-bp insertion (target site 1) and a 155-bp deletion (target site 2) in the other allele (Fig. 3B, Fig. S6). *Mtrgs1*-L10 is a biallelic mutant with a 1-bp deletion (target site 1) and a 6-bp deletion (target site 2) in one allele and a 1-bp deletion (target site 1) and a 7-bp deletion (target site 2) in the other allele; *Mtrgs1*-L11 harbors biallelic mutations with a 4-bp deletion (target site 1) and a 5-bp deletion (target site 2) in one allele and a 4-bp deletion (target site 1) and a 7-bp deletion (target site 2) in the other allele (Fig. 3C, Fig. S6). Each editing event led to frameshift mutations and introduced premature stop codons. Sanger sequencing of PCR products covering potential off-target sites revealed no off-target mutations (Figs. S7–S10). Importantly, the *Mtgβ1* mutants were viable, like the *agb1* mutant of Arabidopsis, and produced fertile seeds, unlike the observations that mutations of *Gβ* in rice, maize, and tomato result in autoimmune phenotypes and seedling death (Gao et al. 2019; Ninh et al. 2021; Utsunomiya et al. 2011; Wu et al. 2020). The above transgenic plants underwent self-pollination and seeds of the T1 generation were used for phenotypic analysis.

We examined seedlings at 7 days after germination, when their cotyledons are open and the first true leaf is emerging. Mutation of *MtGα1* resulted in broader, rounder cotyledons and larger single leaves compared to those of wild-type seedlings. By contrast, the *Mtgβ1* mutants had shorter, rounder cotyledons than the wild type (Fig. 3D). The trifoliate compound leaves of *M. truncatula* begin to emerge at 7 days post-seed germination. When we inspected the third compound leaves of 4-week-old seedlings, the *Mtgα1* and *Mtgβ1* mutants all displayed slightly curly or round leaflets, phenotypes similar to those of the *gpa1* and *agb1* mutants in Arabidopsis, respectively (Ullah et al. 2003). The *Mtgβ1* mutants produced smaller leaves, with fewer marginal protrusions, and the terminal leaflet of the compound leaf structure partially overlapped with the lateral leaflets (Fig. 3E). The loss-of-function mutation of *MtRGS1* did not appear to alter leaf morphology (Fig. 3D, E).

### *MtGα1* and *MtGβ1* affect plant architecture

**Fig. 4:**
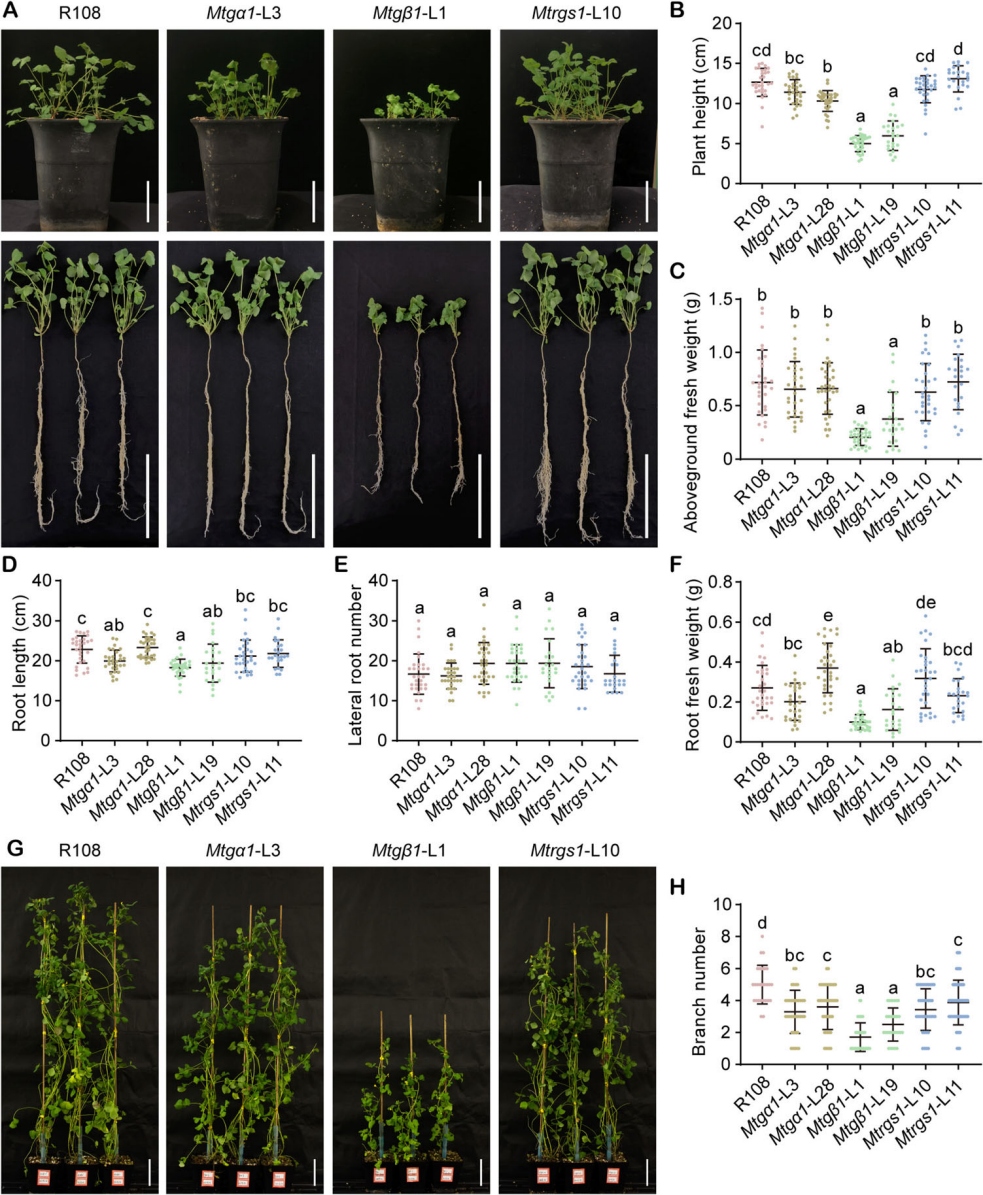
Characterization of the growth and developmental phenotypes of G-protein mutants in *M. truncatula* under normal conditions. **A** Plant architecture of R108, *Mtgα1*, *Mtgβ1* and *Mtrgs1* plants at 4 weeks post-germination. In the lower panel, individual plants were removed from the pots and their roots washed before taking photographs of the root system. Scale bars, 10 cm. **B**–**F** Plant height (**B**), aboveground fresh weight (**C**), root length (**D**), lateral root number (**E**), and root fresh weight (**F**) of R108, *Mtgα1*, *Mtgβ1* and *Mtrgs1* plants. The horizontal lines represent the means (wider line) and the SD range; each dot represents an individual data point. Different lower-case letters indicate statistically significant differences, as determined by a one-way analysis of variance (ANOVA) followed by Tukey’s multiple comparison test. *n* ≥ 22, *P* < 0.01. **G** Representative photographs of R108, *Mtgα1*, *Mtgβ1*, and *Mtrgs1* plants at 8 weeks post-germination. Scale bars, 10 cm. **H** Branch numbers for R108, *Mtgα1*, *Mtgβ1*, and *Mtrgs1* plants at 8 weeks post-germination. The horizontal lines represent the means and the SD range; each dot represents an individual data point. The statistical significance of differences was determined by a one-way ANOVA followed by Tukey’s multiple comparison test; different lower-case letters indicate significant differences. *n* ≥ 27, *P* < 0.01

Plant architecture, encompassing plant height, branch number, branch angle, leaf structure, and root configuration, greatly influences plant biomass and yield potential. Hence, we measured these key traits related to plant architecture in wild-type R108 and the *Mtgα1*, *Mtgβ1*, and *Mtrgs1* mutants. Four weeks post-germination, the *Mtgβ1* mutant lines exhibited a compact stature (Fig. 4A), with plants being shorter by 52–60% relative to wild-type R108 (Fig. 4B) and having 47–70% lower aboveground biomass (Fig. 4C), 15–20% shorter roots (Fig. 4D), and 40–63% lower fresh root weight (Fig. 4F). The *Mtgα1* mutants showed a modest drop in plant height of 9–19%, whereas the *Mtrgs1* mutants maintained a normal plant height, similar to that of R108 (Fig. 4B). The *Mtgα1* and *Mtrgs1* mutants accumulated comparable aboveground and fresh root biomass, had similar root length and lateral root number to R108 plants (Fig. 4C–F). We counted the number of branches at 8 weeks post-germination, observing that all mutants had significantly fewer branches than R108 (Fig. 4G, H). These results indicate that *MtGα1* and especially *MtGβ1* shape plant architecture in *M. truncatula*.

**Fig. 5:**
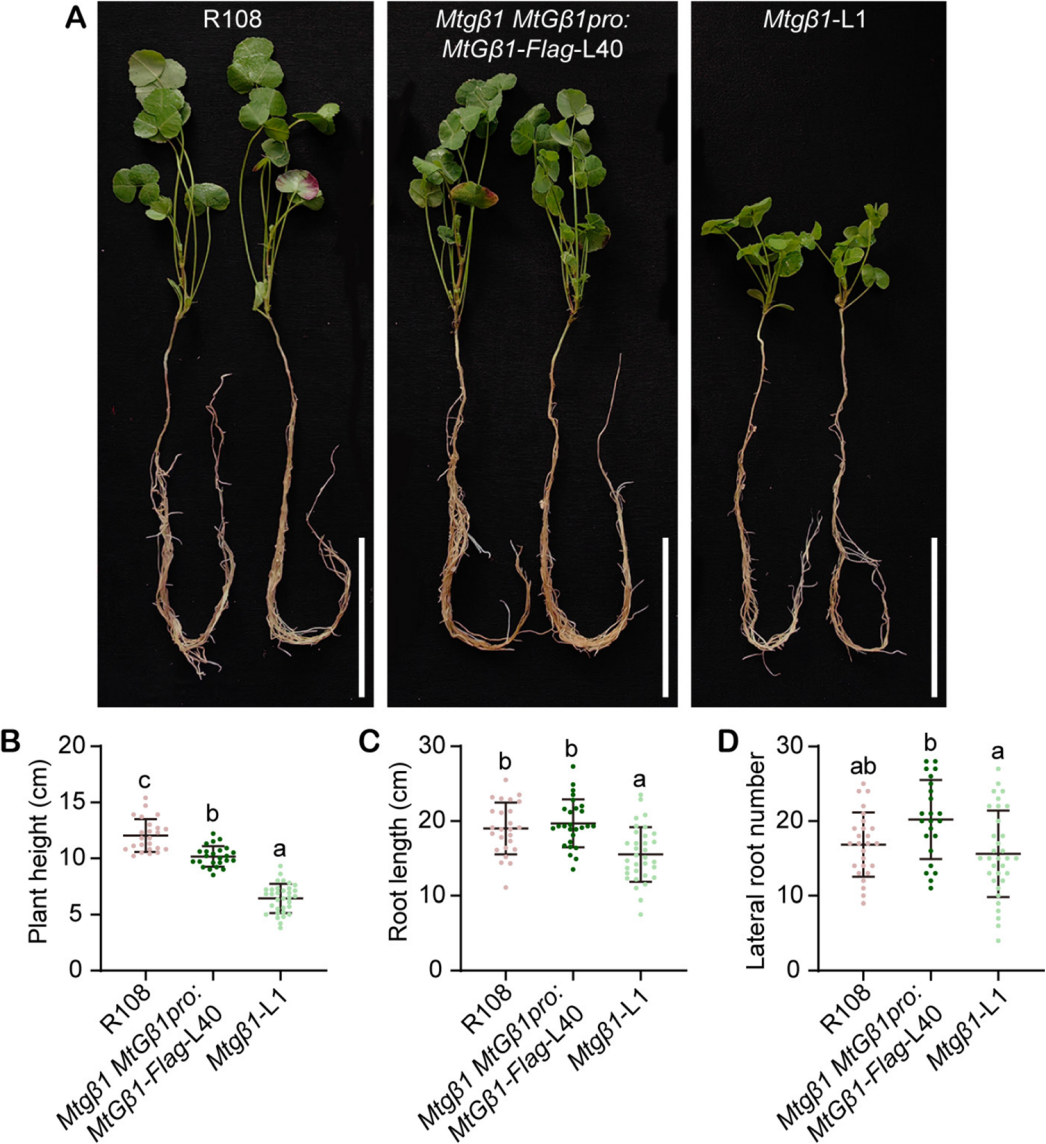
Genetic complementation of *MtGβ1.* **A** Plant architecture of R108, *Mtgβ1 MtGβ1pro:MtGβ1-Flag*-L40 and *Mtgβ1*-L1 plants at 4 weeks post-germination. Scale bar, 10 cm. **B**–**D** Plant height (**B**), root length (**C**), and lateral root number (**D**) of the genotypes shown in **A**. The horizontal lines represent the means (wider line) and the SD range; each dot represents an individual data point. Different lower-case letters indicate statistically significant differences, as determined by a one-way ANOVA followed by Tukey’s multiple comparison test. *n* ≥ 23, *P* < 0.01

To verify that the developmental defects observed in *Mtgβ1* mutant lines were indeed caused by the mutation of *MtGβ1*, we constructed the complementation construct *MtGβ1pro:MtGβ1-Flag*, consisting of a 3013-bp *MtGβ1* promoter fragment and the 1134-bp *MtGβ1* coding sequence cloned in-frame and upstream of the sequence for a Flag tag. We then introduced this construct into Cas9-free *Mtgβ1*-L1 mutant T2 generation plants by Agrobacterium-mediated transformation. We allowed several regenerated T0 plants to undergo self-pollination and collected seeds for the T1 generation. After sowing these seeds, we characterized the resulting plants by genotyping PCR, RT-qPCR, and immunoblot analysis (Fig. S11), as well as phenotypic analysis. The T2 plants harboring the complementation construct were significantly taller and had significantly longer roots than *Mtgβ1*-L1 plants at 4 weeks post-germination, with root length returning to R108 values (Fig. 5), confirming that MtGβ1 influences growth and development.

The rice mutant in *Gα*, dwarf 1 (*d1*) has dark-green leaves, and the *d1* mutant and plants overexpressing *RGG1* or *RGB1* possess higher photosynthetic capacity (*F*_v_*/F*_m_) and enhanced drought tolerance compared to the wild type (Swain et al. 2019; Zait et al. 2021). However, we did not observe differences in the leaf color of the mutant plants relative to those of the wild type (Fig. 4A). We also measured photosynthetic efficiency indices in R108 and the *Mtgα1*, *Mtgβ1*, and *Mtrgs1* mutants. The mutants had similar maximum quantum yields (*F*_v_*/F*_m_) (Fig. S12A), actual quantum yields (II) (Fig. S12B), and electron transport rates (ETR) (Fig. S12C) to R108 plants, suggesting that G proteins might play a different role in stress tolerance in *M. truncatula* than in rice, specifically in drought tolerance.

### *MtGα1* and *MtGβ1* regulate pod development and seed size

**Fig. 6:**
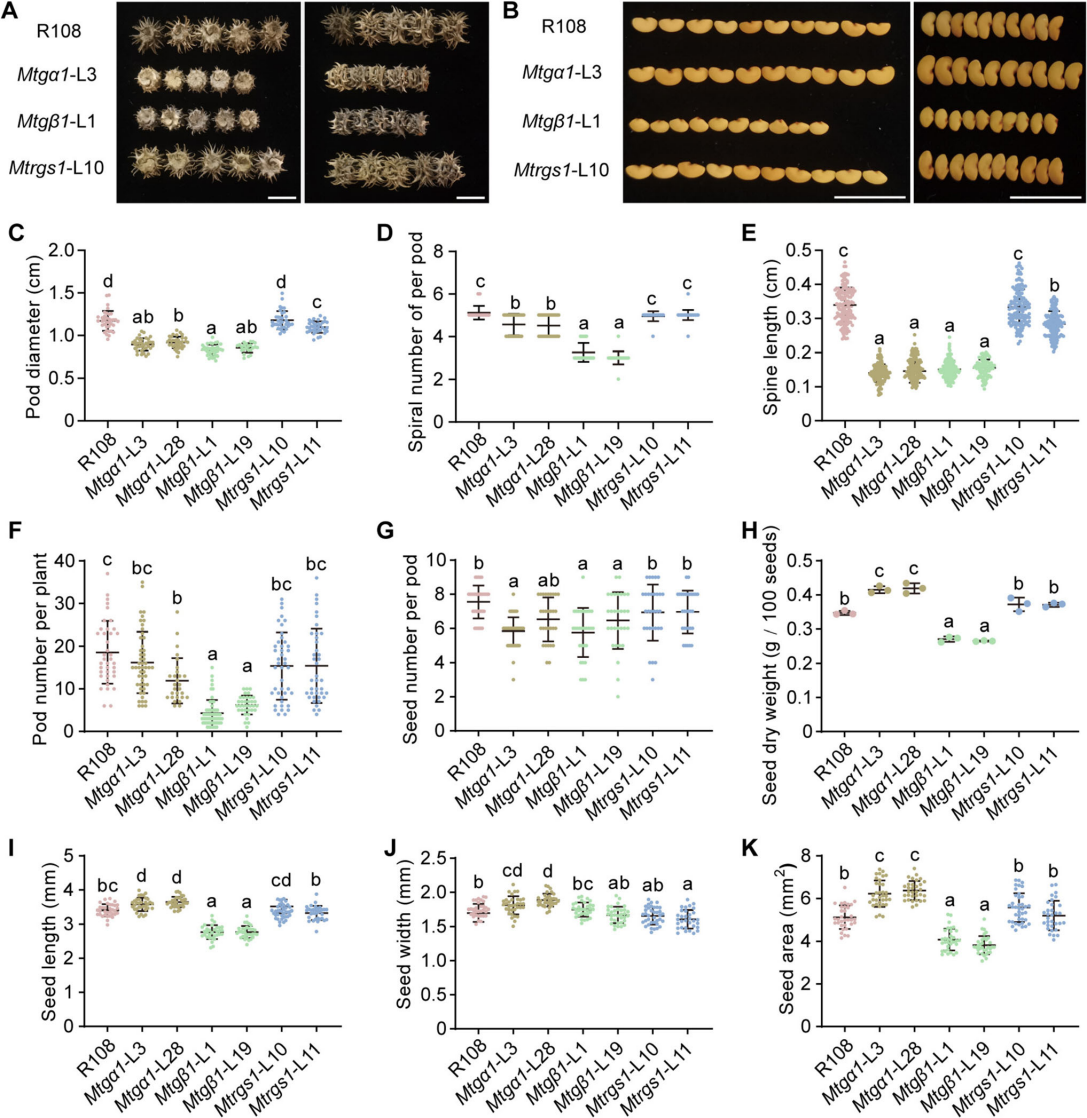
Pod and seed phenotypes of G-protein mutants in *M. truncatula* under normal conditions. **A**, **B** Representative photographs of pods (**A**) and seeds (**B**) from R108, *Mtgα1*, *Mtgβ1* and *Mtrgs1* plants. Scale bars, 1 cm. **C**–**K** Pod diameter (**C**), spiral number of per pod (**D**), spine length (**E**), pod number per plant (**F**), seed number per pod (**G**), seed dry weight (**H**), seed length (**I**), seed width (**J**), and seed area (**K**) from R108, *Mtgα1*, *Mtgβ1* and *Mtrgs1* plants. The horizontal lines represent the means (wider line) and the SD range indicated; each dot represents an individual data point. Different lower-case letters indicate statistically significant differences, as determined by a one-way ANOVA followed by Tukey’s multiple comparison test. *n* ≥ 22 (**C**–**G**, **I**–**K**) and *n* = 3 (**H**). *P* < 0.01

The seed pod is the most prevalent and basic fruit type in the extensive Leguminosae family. *Medicago* pods develop into a coiled, barrel-like structure with surfaces covered by curved or hooked spines, which may attach to passing animals, thereby facilitating long-distance dispersal (Roque et al. 2018). We measured the pod diameter and spine length and counted the number of spirals of mature seed pods. Loss-of-function mutations of *MtGα1* or *MtGβ1* led to smaller pods with fewer spiral turns and shorter spines than the wild type, with a more severe defect in the number of spiral turns seen in the *Mtgβ1* mutants. By contrast, the pods of the *Mtrgs1* mutants were comparable to those of R108 (Fig. 6A, C–E). These results suggest that *MtGα1* and *MtGβ1* might have analogous roles in pod development.

In addition, the size of seed pods, seed size is a primary agronomic trait in crop plants. Compared to R108, the *Mtgα1* mutant lines produced larger seeds, with the hundred-seed weight being greater by 19–21%, whereas the *Mtgβ1* mutant lines had smaller seeds, with a hundred-seed weight dropping by 22–24% relative to R108 (Fig. 6B, H–K). We also counted the total number of pods on each plant and the number of seeds per pod. The *Mtgβ1* mutants bore 66–77% fewer pods per plant, with each pod containing 14–24% fewer seeds relative to R108, whereas the *Mtgα1* mutants exhibited minor declines in these metrics (Fig. 6F, G). However, the *Mtrgs1* mutants were similar to R108 in terms of pod development, seed size, and yield potential (Fig. 6). These results suggest that both MtGα1 and MtGβ1 have important functions in modulating reproductive growth, although their regulatory effects on pod development and seed size might differ.

### *MtGβ1* positively regulates nodulation

**Fig. 7:**
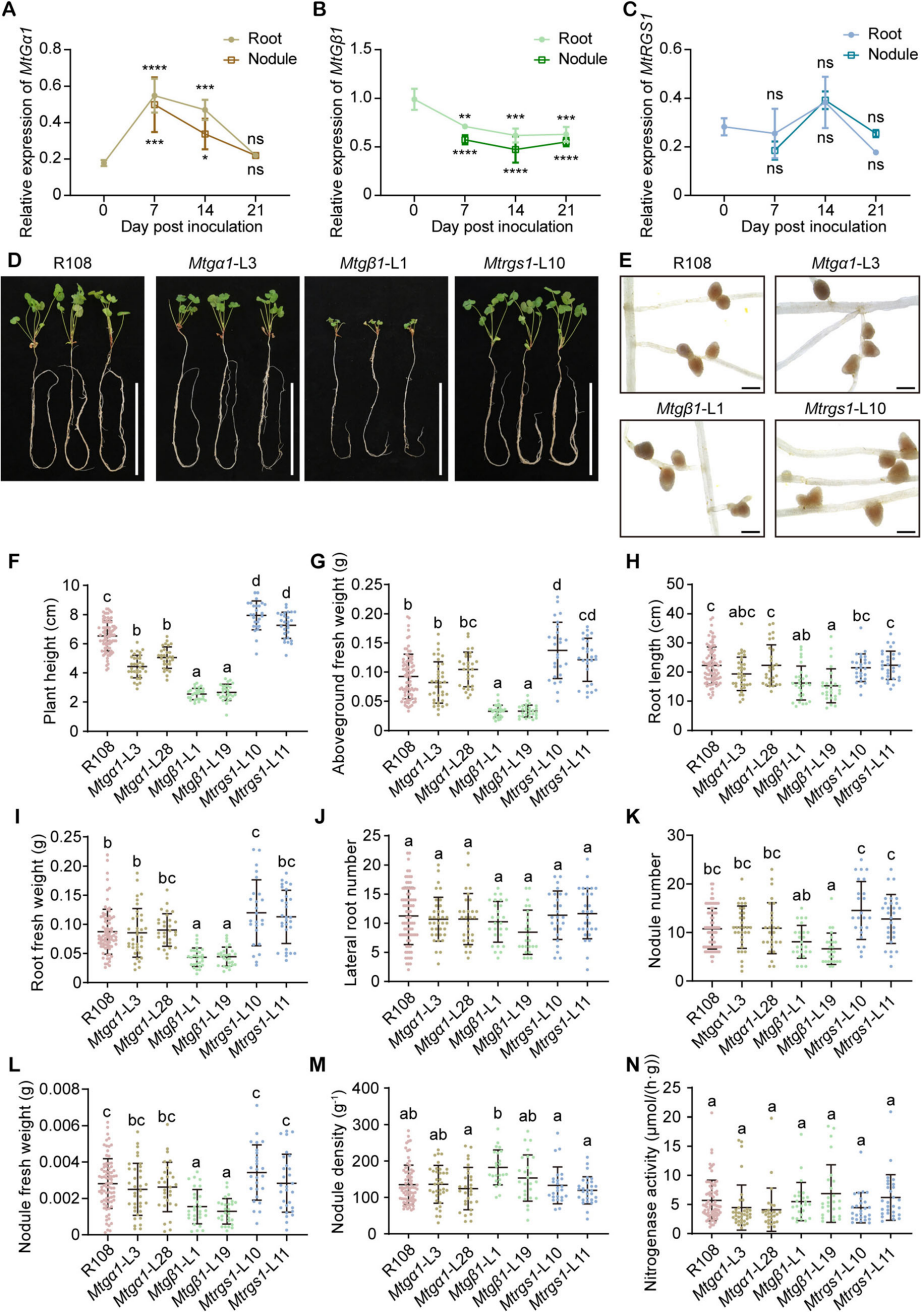
Nodulation phenotypes of G-protein mutants in *M. truncatula* after inoculation with Sm1021. **A**–**C** RT-qPCR analysis of the expression levels of *MtGα1* (**A**), *MtGβ1* (**B**), and *MtRGS1* (**C**) in roots of R108 seedlings at 0, 7, 14, and 21 days post-inoculation (dpi), and nodules at 7, 14, and 21 dpi. The expression level of each gene was normalized to those of *MtACTIN11* and *MtRBP1*. Values are means ± SD from three biological replicates. The statistical significance of differences was determined by a one-way ANOVA; the asterisks indicate the significance of differences at each time point compared to the 0-dpi sample. ns, *P* ≥ 0.05; *, *P* < 0.05; **, *P* < 0.01; ***, *P* < 0.001; ****, *P* < 0.0001. **D**, **E** Representative photographs of whole plants (**D**, scale bar, 10 cm) and nodules (**E**, scale bar, 1 mm) of R108, *Mtgα1*, *Mtgβ1*, and *Mtrgs1* plants at 21 dpi. **F**–**N** Plant height (**F**), aboveground fresh weight (**G**), root length (**H**), root fresh weight (**I**), lateral root number (**J**), nodule number (**K**), nodule fresh weight (**I**), nodule density (**M**), and nitrogenase activity (**N**) of R108, *Mtgα1*, *Mtgβ1* and *Mtrgs1* plants. The horizontal lines represent means, with the SD range indicated; each dot represents an individual data point. Different lower-case letters indicate statistically significant differences, as determined by a one-way ANOVA followed by Tukey’s multiple comparison test. *n* ≥ 24, *P* < 0.01

The capacity of legumes to establish a symbiotic relationship with nitrogen-fixing bacteria is ecologically advantageous and crucial for crop yields (Fang et al. 2024). The *Medicago*–*Sinorhizobium* symbiotic system stands as one of the best characterized models of legume–rhizobium interactions, especially in the context of indeterminate nodule formation (Berrabah et al. 2018). To elucidate the possible contributions of G proteins to symbiotic nodulation in *M. truncatula*, we used RT-qPCR to examine the expression levels of *MtGα1*, *MtGβ1*, and *MtRGS1* in the roots of R108 plants at 0, 7, 14, and 21 days post-inoculation (dpi) with Sm1021, as well as in nodules at 7, 14, and 21 dpi. The expression patterns of each gene in nodules were similar to those in roots. The expression of *MtGα1* reached a peak at 7 dpi in both tissues (Fig. 7A). *MtGβ1* expression levels declined significantly following inoculation (Fig. 7B). Additionally, *MtRGS1* expression appeared to dropped at 7 dpi and then rise at 14 dpi, although the differences were not statistically insignificant (Fig. 7C). These results suggest that MtGα1, MtGβ1, and MtRGS1 might participate in symbiotic nodulation of *M. truncatula*.

We performed phenotypic assays to evaluate the symbiotic nodulation and nitrogen fixation capacities of the G-protein mutants 21 days after inoculation with Sm1021. Following inoculation, *Mtgβ1* plants were shorter by 59–61% (Fig. 7D, F), accumulated 64–65% less aboveground biomass (Fig. 7G), had 27–31% shorter roots (Fig. 7H), and had 49–50% lighter roots (in terms of fresh weight; Fig. 7I) relative to wild type R108, but maintained the same number of lateral roots (Fig. 7J). These results largely aligned with the phenotypic differences observed under normal growth conditions between the wild type and the mutants (Fig. 4B–F). Notably, the mutation of *MtGβ1* led to 28–38% fewer nodules (Fig. 7K), a difference that reached statistical significance in the *Mtgβ1*-L19. Although the mutation of *MtGβ1* did not affect nodule density (Fig. 7M) or nitrogenase activity (Fig. 7N), it significantly diminished nodule fresh weight (Fig. 7L), which could lead to a lower total nitrogen-fixing capacity. The *Mtgα1* mutant plants were 23–32% shorter than R108 plants at 21 dpi (Fig. 7D, F), aligning with the developmental phenotypic trend seen with *Mtgα1* under normal growth conditions (Fig. 4), and produced a similar number of nodules, nodule fresh weight, nodule density, and nitrogenase activity to wild-type plants (Fig. 7K–N). In addition, the *Mtrgs1* mutants showed normal root and nodule phenotypes that were indistinguishable from those of R108 (Fig. 7H–N). These results indicate that MtGβ1 plays a role modulates development and symbiotic nodulation in *M. truncatula*, whereas MtGα1 and MtRGS1 might be less important to these processes.

## Discussion

In this study, we functionally characterized the genes encoding the G-protein subunits Gα and Gβ, as well as their regulator RGS, in the model legume *M. truncatula*. The composition of the core canonical G proteins in *M. truncatula* appear to be relatively simple, and similar to those in Arabidopsis, rice, and maize, with only one *Gα* and one *Gβ* genes. *MtGα1*, *MtGβ1*, and *MtRGS1* were widely expressed in multiple tissues, suggesting they exert functions at various stages of growth and development. The subcellular location of MtGα1 and MtRGS1 at the plasma membrane suggests a function in receiving and transducing signals from the environment, whereas the presence of MtGβ1 at the plasma membrane, cytoplasm, and nucleus aligns with the fact that Gβ can associate with Gα to form heterotrimers at the plasma membrane, but can also function independently of Gα in the cytoplasm and nucleus.

Although the mutant database available for *M. truncatula* lists mutants in nearly all genes in the genome and offers invaluable genetic resources for gene functional analysis (Sun et al. 2018, 2019; Tadege et al. 2008), mutants of G-protein genes have yet to be identified in either the fast neutron bombardment deletion mutant library or the Tnt1 retrotransposon insertion library, which impeded a detailed analysis of G-protein functions until now. Here, we employed reverse genetics to elucidate the functions of G proteins in *M. truncatula*. Specifically, we designed CRISPR/Cas9 constructs and successfully obtained *Mtgα1*, *Mtgβ1*, and *Mtrgs1* knockout mutants via Agrobacterium-mediated transformation. Their phenotypic analysis revealed that *MtGα1* and *MtGβ1* play important roles in plant growth and development, whereas *MtRGS1* contributes to these processes only to a relatively modest degree. These mutants, which display complex phenotypes, serve as important genetic resources for conducting in-depth investigations into the underlying mechanisms of G proteins.

Mutation of *MtGα1* in *M. truncatula* also produced a range of phenotypic effects, some of them similar to those of *gpa1* in Arabidopsis, *d1* in rice, and *ct2* in maize, such as curly, round leaflets, and slight dwarfism (Figs. 3E, 4A, B, G) (Bommert et al. 2013; Fujisawa et al. 1999; Ullah et al. 2001; Urano et al. 2015), whereas others were quite distinctive. For example, *Mtgα1* mutants formed smaller pods, yet larger and heavier seeds (Fig. 6A–D, H–K), a phenomenon markedly different from observations in rice and *Brassica juncea*, where the knockout or knockdown of *Gα* led to smaller seeds (Kumar and Bisht 2020; Oki et al. 2009). In addition, the loss-of-function mutants of *MtGα1* did not affect root architecture, whereas the genetic inactivation of *Gα* in Arabidopsis, rice, and maize inhibited root development (Chen et al. 2006a; Izawa et al. 2010; Ullah et al. 2003; Urano et al. 2015).

*Mtgβ1* mutants displayed an overall smaller plant architecture (Fig. 4A), including smaller leaflets (Fig. 3E), shorter plants (Fig. 4B), fewer branches (Fig. 4H), lower biomass (Fig. 4C), and smaller pods with shorter spines and smaller, lighter seeds (Fig. 6); similar observations have been reported for the corresponding mutants in Arabidopsis and rice (Chen et al. 2006a; Lease et al. 2001; Trusov et al. 2008; Ullah et al. 2003; Utsunomiya et al. 2011). In particular, *Mtgβ1* mutants had markedly shorter (Fig. 4D) and lighter roots (Fig. 4F), in contrast to the Arabidopsis *agb1* mutant, which has more lateral roots with greater root biomass (Trusov et al. 2008). However, in monocot species such as rice and maize, and in certain dicot species such as tomato and cotton (*Gossypium hirsutum*), mutation of *Gβ* leads to seedling death (Gao et al. 2019; Ninh et al. 2021; Utsunomiya et al. 2011; Wu et al. 2020), indicative of variations in Gβ functions among different species.

*RGS* is absent from the genome of numerous plant species, indicating that it may be a species-specific conditional regulator of the G-protein cycle. The *Mtrgs1* mutants we obtained in this research will be useful for further understanding the regulatory network of G proteins in *M. truncatula*. There were no significant differences in growth or development under normal conditions between the *Mtrgs1* mutants and the wild-type R108 at the early stages of growth (4 weeks post germination). However, in the later stages, we noticed a slight impairment in the *Mtrgs1* mutants, as evidenced by fewer branches. Previous studies in Arabidopsis have shown that the loss of *AtRGS1* is accompanied with weak phenotypes under static conditions, but is associated with strong phenotypes when dynamic signals exist, such as light and flg22 (Ghusinga et al. 2022; Liang et al. 2018; Liao et al. 2017). Photosynthesis efficiency in *rgs1* mutants is reduced in a changing-but not a constant-light environment (Liao et al. 2017). The *rgs1* mutants display significantly increased H_2_O_2_ production in response to flg22 or chitin treatment (Liang et al. 2018). These observations suggest that RGS proteins might modulate G-protein signaling networks in response to stimuli, while exerting minimal influence on growth and development.

From the perspective of plant reproductive stages, *Mtgα1* and *Mtgβ1* mutants both bore smaller seed pods decorated with shorter spines as compared to the wild type. *M. truncatula* plants overexpressing the precursor for the microRNA MtmiR156B, which targets the transcripts of several *SQUAMOSA Promoter-Binding Protein-Like* (*MtSPL*) genes highly expressed in seed pods, were reported to bear smaller pods without spines (Wang et al. 2019). OsSPL5 was recently shown to bind to the promoter of *DENSE AND ERECT PANICLE 1* (*DEP1*), encoding a Gγ subunit, resulting in higher *DEP1* expression, thereby modulating stigma and panicle development in rice (Li et al. 2024). The seed pod and spine phenotypes of the *Mtgα1* and *Mtgβ1* mutants were similar to those observed in plants overexpressing *MtmiR156B*, suggesting that the miR156B–SPL module might regulate the expression of G-protein subunit genes to modulate seed pod development. Another smooth pod mutant of *M. truncatula*, *seed smooth pod 1* (*ssp1*), was obtained during tissue culture and was attributed to a mutation in a gene encoding the immunophilin-like FK506-binding protein 42 (FKBP42, also reported as TWISTED DWARF 1 [TWD1] in Arabidopsis). *SSP1* showed similar expression patterns to the auxin output promoter *DR5* during spine formation, suggesting the participation of auxin transport at this stage (Zhao et al. 2021). The pod spines of the *Mtgα1* and *Mtgβ1* mutants displayed similarities to those observed in plants lacking SSP1 function, suggesting that MtGα1 and MtGβ1 might influence auxin transport during spine development.

Another fascinating finding is that despite the smaller seed pods of *Mtgα1* mutants, their seeds were larger. Several studies have revealed how the STERILE APETALA–PEAPOD–KINASE-INDUCIBLE DOMAIN INTERACTING–TOPLESS (SAP–PPD–KIX–TPL) module plays a conserved role in regulating organ size in dicot species. The *PPD* orthologous gene *BIG SEED 1* (*BS1*) in *M. truncatula*, which encodes a class II member of the TIFY family of transcriptional regulators, controls the size of plant organs, including seeds, seed pods, and leaves, by negatively regulating primary cell proliferation (Ge et al. 2016). MtKIX8 acts as an adaptor between BS1 and the co-repressor MtTPL to repress organ size, whereas the *M. truncatula* F-box protein MINI ORGAN1 (MIO1), an ortholog of Arabidopsis SAP, forms an SCF E3 ubiquitin ligase complex to target substrate proteins for degradation, positively regulating organ size (Mao et al. 2023; Zhou et al. 2021). Mutations of *BS1* or *MtKIX8*, or the overexpression of *MIO1*, results in larger seeds (Ge et al. 2016; Mao et al. 2023; Zhou et al. 2021), reminiscent of the phenotypes observed in the *Mtgα1* mutants. In addition, seed pods, seeds, and leaves were all significantly smaller in the *Mtgβ1* mutants. Therefore, we hypothesize that mutation of *MtGa1* may free MtGβ1 to suppress downstream negative regulators involved in seed development, such as the MIO1–BS1–KIX8 module, yielding larger seeds. However, the *Mtgα1* mutants displayed diverse patterns in the sizes of various organs, particularly the seed pods and seeds, suggesting that MtGα1 might regulate the development of seed pods and seeds via different modes, possibly involving distinct effector proteins.

Previous research has investigated the involvement of G proteins in the regulation of symbiotic nodulation in soybean and the role of MtGβ1 in the A17 accession of *M. truncatula*. In this study, we assessed the phenotypic consequences of mutations in G-protein genes on nodulation in R108 of *M. truncatula*, and noted similarities and discrepancies between our results and those reported previously. The expression levels of *MtGα1* rose in both A17 and R108 during the early stages after inoculation, before declining back to a baseline pre-inoculation level (Fig. 7A) (Schiessl et al. 2019). In contrast, *MtGβ1* expression decreased after inoculation in both A17 and R108 (Fig. 7B) (Schiessl et al. 2019). These results suggest that G proteins might play a similar role in these two *M. truncatula* ecotypes. In A17, the RNAi-based silencing of *MtGβ1* resulted in significantly fewer nodules at 14 dpi in a previous study (Bovin et al. 2022). We observed a similar outcome with our *Mtgβ1* mutants in the R108 background at 21 dpi compared to wild-type R108, with the smaller nodule number reaching statistical significance in *Mtgβ1*-L19 plants (Fig. 7K). Moreover, the *Mtgβ1* mutants displayed a marked drop in nodule fresh weight in this study (Fig. 7L). The difference of nodule number between the two ecotypes may be related to the distinct analysis time used in the two studies. In conclusion, these observations suggest that MtGβ1 positively regulates nodulation in the A17 and R108 ecotypes of *M. truncatula*.

Regarding the role of Gα and RGS in nodulation, the *Mtgα1* and *Mtrgs1* mutants did not show significant alterations in their root or nodule phenotypes. In soybean, phosphorylation of RGS proteins by NFR1 and SymRK enhance their activity to accelerate GTP hydrolysis by Gα proteins, thereby positively regulating nodulation (Choudhury and Pandey 2015, 2024). Based on multiple protein sequence alignments, we noticed that the phosphorylation site of serine 269 in GmRGS2, a target site of phosphorylation by NFR1 and SymRK (Choudhury and Pandey 2015, 2024), corresponds to a nonphosphorylatable glycine residue in MtRGS1. This change may lead to lower MtRGS1 phosphorylation, potentially affecting its GTPase-accelerating activity. Additionally, nodules in *M. truncatula* are classified as of the indeterminate type, characterized by the presence of a persistent apical meristem, and thus different from the determinate nodules of soybean. The distinction in the formation of indeterminate and determinate nodules is related to the initial buildup of an auxin maximum in different layers of the cortex (Kohlen et al. 2018), and the absence of the apical meristem in determinate nodules is attributed to the differential distribution of components in the NODULE INCEPTION (NIN)–auxin feedback loop (Tu et al. 2024). The G-protein interactor N-MYC DOWNREGULATED-LIKE (NDL) proteins NDL1 to NDL3 were reported to interact with Gβγ to modulate primary root length and lateral root density by affecting the expression of auxin transporter proteins in Arabidopsis (Mudgil et al. 2009). The potential functions of G proteins in regulating auxin transport might vary between the formation of indeterminate and determinate nodules in different legumes. In summary, the G-protein signaling network may have a distinct regulatory mechanism in the context of determinate versus indeterminate nodulation.

**Fig. 8:**
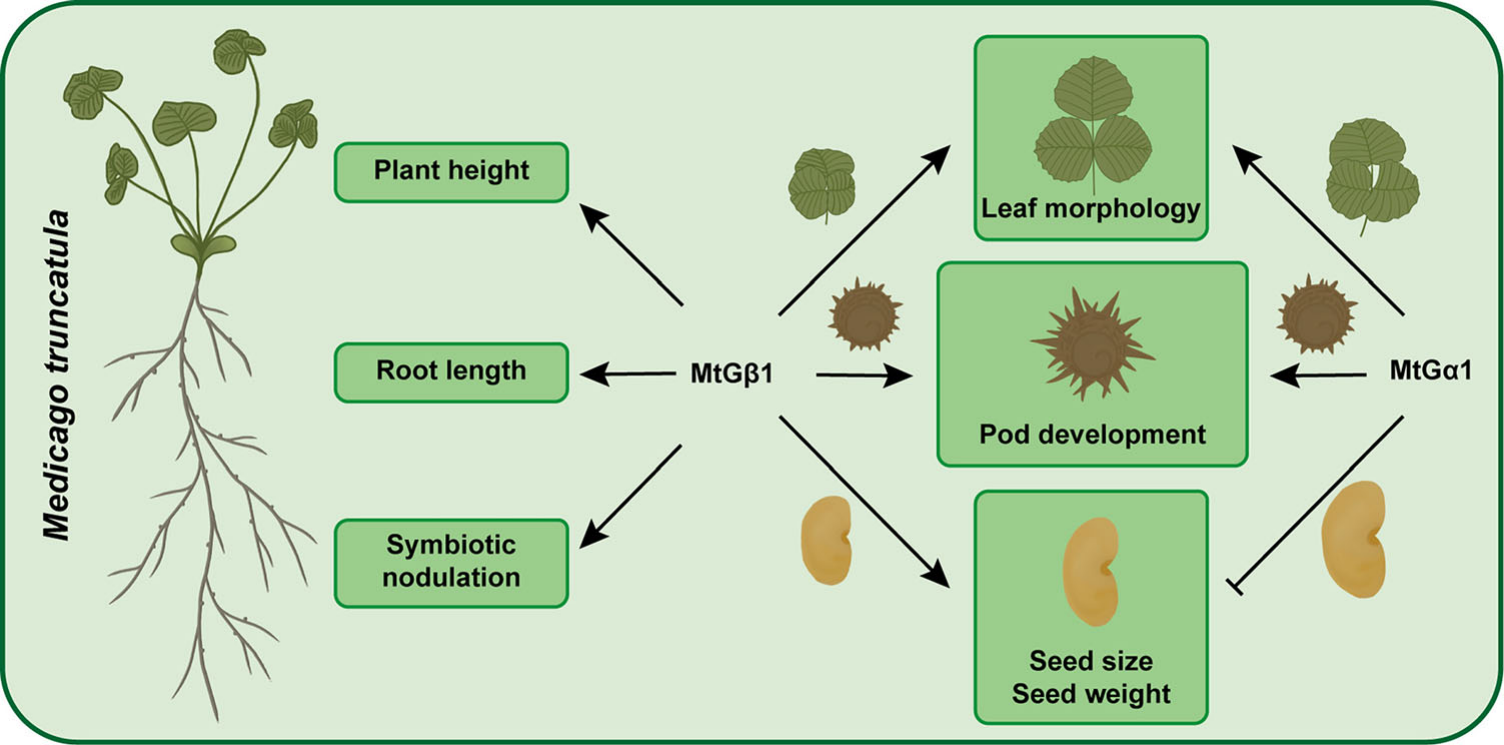
Summary of traits affected by MtGα1 and MtGβ1 in *M. truncatula*. MtGβ1 plays an important role in plant growth and development. MtGβ1 positively regulates plant height, root length, symbiotic nodulation, leaf size, leaf marginal protrusions, pod development, seed size and seed weight. MtGα1 positively regulates pod development, negatively regulates seed size and seed weight, and affects leaf morphology. Positive or negative regulations are denoted by lines ending with an arrow head or bar, respectively

Collectively, in *M. truncatula*, MtGβ1 plays important roles in regulating plant height, root length, symbiotic nodulation, leaf size, leaf marginal protrusions, pod development, seed size and seed weight. MtGα1 positively regulates pod development, negatively regulates seed size and seed weight, and affects leaf morphology (Fig. 8). The phenotypes of G-protein mutants in *M. truncatula* display both similarities and differences when compared to those in other plant species, highlighting the inherent complexity of G-protein signaling pathways. Our research broadens the understanding of the biological functions of G proteins in a model legume and establishes a foundation for further investigations into their intricate molecular mechanisms.

## Materials and methods

### Plant materials and growth conditions

The *M. truncatula* R108 accession was used as the wild type in this study. Seeds were immersed in 98% (v/v) H_2_SO_4_ for 8 min, washed three times with ice-cold water, surface-sterilized with 0.5% (w/v) NaClO for 10 min, and washed six times with sterile deionized water. After this pre-treatment, the seeds were placed on the surface of 0.8% (w/v) water agar medium and stratified at 4 °C in darkness for 3 days. The seeds were then allowed to germinate at 24 °C under dark conditions for 16 h. The newly germinated seedlings were subsequently planted to a mixture of vermiculite:perlite (5:2, v/v) saturated with different medium in a greenhouse as followed.

Seedlings and plants were grown in a greenhouse at 22 °C, under a 16-h light/8-h dark photoperiod (light intensity of 100–150 μmol m^−2^ s^−1^) and a relative humidity of 60–70%. For normal growth and reproduction, plants were grown in a mixture of vermiculite:perlite (5:2, v/v) saturated with half-strength liquid Murashige and Skoog (MS) medium (Phyto Technology, cat# M519). For nodulation, plants were grown in a mixture of vermiculite:perlite (5:2, v/v) soaked in nitrogen-free liquid Fåhraeus medium for 7 days and then inoculated with the rhizobium *Sinorhizobium meliloti* strain 1021 (Sm1021) resuspended in nitrogen-free liquid Fåhraeus medium (OD at 600 nm, 0.05). The rhizobium strain Sm1021 was cultured in TY medium.

### Identification of homologs and phylogenetic analysis

The protein sequences of Gα, Gβ, Gγ, and RGS were obtained from Phytozome databases (https://phytozome-next.jgi.doe.gov/) using BLAST tools. The protein sequences of Gα, Gβ, and Gγ from Arabidopsis and rice were used as queries, and the protein sequences of RGS from Arabidopsis were used as queries. Multiple protein sequence alignments were performed using MUSCLE algorithm (Edgar 2004). The phylogenetic trees were generated by the neighbor-joining method using MEGA 11 software. The trees were evaluated with 1000 bootstrap replicates.

### CRISPR/Cas9 vector construction and complementation test

The knockout mutants *Mtgα1*, *Mtgβ1*, and *Mtrgs1* were generated by CRISPR/Cas9-mediated gene editing. Two target sites for each gene were selected via the website tool CRISPOR (http://crispor.tefor.net/) (Concordet and Haeussler 2018) and the corresponding single guide RNA (sgRNA) sequences were cloned into the binary vector p6401 conferring resistance to hygromycin in transgenic plants (Zhu et al. 2021). The resulting constructs were separately introduced into Agrobacterium (*Agrobacterium tumefaciens*) strain EHA105 for transformation of R108 plants via Agrobacterium-mediated transformation. The transformation method was described in Xue et al. (2023). The regenerated transgenic T0 seedlings were identified by PCR and the mutation types were identified by Sanger sequencing. In addition, PCR products for all potential off-target sites predicted by CRISPOR were subjected to Sanger sequencing. The primers used in this experiment are listed in Table S1.

To generate complementation constructs, a 4240-bp fragment containing the entire *MtGβ1* coding sequence and a 3013-bp upstream promoter sequence was amplified from R108 cDNA, to which the sequence for a Flag tag was added before cloning into the pCAMBIA1381 vector conferring resistance to hygromycin in transgenic plants. The resulting plasmid was introduced into Cas9-free *Mtgβ1*-L1 T2 homozygous mutant plants via Agrobacterium (strain EHA105)-mediated transformation. The Cas9-free *Mtgβ1*-L1 T1 were indentified using MtGβ1-Target1-F0 and MtGβ1-Target2-R0 primers, and these T1 plants underwent self-pollination and seeds of the T2 generation plants were used for constructing complementation plants. The regenerated complementation seedlings were tested by PCR, RT-qPCR, and immunoblot analysis. The primers used in this experiment are listed in Table S1.

### RNA extraction and RT-qPCR analysis

RT-qPCR was used to detect the transcript levels in different tissues or in response to rhizobial inoculation. The root and leaf samples were collected from 4-week-old wild-type plants, and the shoot, flower, and pod samples were collected from 8–10-week-old wild-type plants. For nodulation analysis, seedlings were inoculated with Sm1021 after nitrogen starvation for 7 days. The roots and nodules were collected at 7, 14, and 21 days post-inoculation (dpi), also collecting roots from non-inoculated seedlings as a 0 dpi control.

Total RNA was extracted with Trizol reagent. Moloney murine leukemia virus (M-MLV) reverse transcriptase (Promega, cat# M1701) was used to synthesize first-strand cDNA. qPCR was carried out on a CFX-96 real-time system (Bio-Rad) with SYBR Premix Ex-Taq (TaKaRa, cat# RR420A). Gene expression levels were calculated using the 2^−ΔΔCT^ method (Livak and Schmittgen 2001) and normalized to *MtACTIN4A* levels in different tissues analysis, or to *MtACTIN11* and *MtRBP1* in nodulation analysis. The primers used in this experiment are listed in Table S1.

### Immunoblot assays

The stable transformed *Mtgβ1 MtGβ1pro:MtGβ1-Flag* lines were used for immunoblot assays. The seedlings were grown under normal greenhouse conditions, and their leaves of 4-week-old T1 plants were collected for protein extraction using the NP-40 Lysis Buffer (Beyotime, cat# P0013F). An anti-Flag monoclonal antibody (Sigma, cat# F1804) was used for detecting the target protein. Actin was used as the loading control and was detected with an anti-Actin antibody (CWBIO, cat# CW0264).

### Subcellular localization and confocal imaging

For the subcellular localization of MtGα1, MtGβ1, and MtRGS1, the full-length coding sequences of *MtGα1*, *MtGβ1* or *MtRGS1* was cloned into the pCAMBIA1307-p35S-cGFP to construct the *35S*:*MtGα1*-*GFP*, *35S:MtGβ1*-*GFP* and *35S:MtRGS1-GFP* plasmids, and the full-length coding sequences of *MtGβ1* was also cloned into the pCAMBIA1307-p35S-nGFP binary vector to construct the *35S:GFP*-*MtGβ1* plasmid. The resulting constructs were introduced individually into Agrobacterium strain EHA105, and bacterial cell suspensions from positive colonies harboring each construct were co-infiltrated into the leaves of *Nicotiana benthamiana* plants with a bacterial cell suspension of Agrobacterium carrying the plasma membrane marker plasmid *35S:AtCBL1n-mCherry*. Two days after infiltration, the infiltrated leaves were infiltrated with 0.5–10 μg/mL 4′,6-diamidino-2-phenylindole (DAPI) (Sigma, cat# 28718-90-3) and subsequently were observed on a Leica SP8 confocal microscope. The excitation and emission wavelengths were 488 nm and 495–545 nm for GFP, 552 nm and 590–670 nm for mCherry, and 409 nm and 380–425 nm for DAPI, respectively.

### Measurement of photosynthetic efficiency

The maximum quantum yield of photosystem II (PSII) efficiency (*F*_v_*/F*_m_), actual quantum yield (II) [Y(II)], and electron transport rate (ETR) were measured using leaves with a MINI-PAM-II (Walz, Germany). Prior to measurements, the leaves of 4-week-old plants were dark-adapted for 30 min.

### Protein structure prediction

Protein domain predictions of Gα, Gβ, and RGS were used by SMART website (https://smart.embl.de/). The AlphaFold 3 webserver (http://golgi.sandbox.google.com/) was used to model the 3D structures of Gα, Gβ, and RGS. The structural models of these proteins were visualized using PyMOL.

### Statistical analysis

The data are presented as means ± standard deviation (SD). Assessments of significance were performed by nonparametric one-way analysis of variance (ANOVA) followed by Tukey’s test using SPSS statistical software. The *P*-values for each statistical test are reported.

## Supplementary Information

Below is the link to the electronic supplementary material.

Supplementary file 1 (DOCX 45594 KB)

## Data Availability

The authors confirm that all data from this study are available and can be found in this article and in the supplementary information.
