# Supplementary material for: Heterotrimeric G-protein subunits regulate plant architecture, pod development, seed size, and symbiotic nodulation in Medicago truncatula
Source: aBIOTECH. 2025 May 7;6(2):141–59. doi: 10.1007/s42994-025-00210-x (PMC12238709; doi:10.1007/s42994-025-00210-x)
Supplement: Supplementary file 1 — (DOCX 45594 KB) [file 42994_2025_210_MOESM1_ESM.docx]

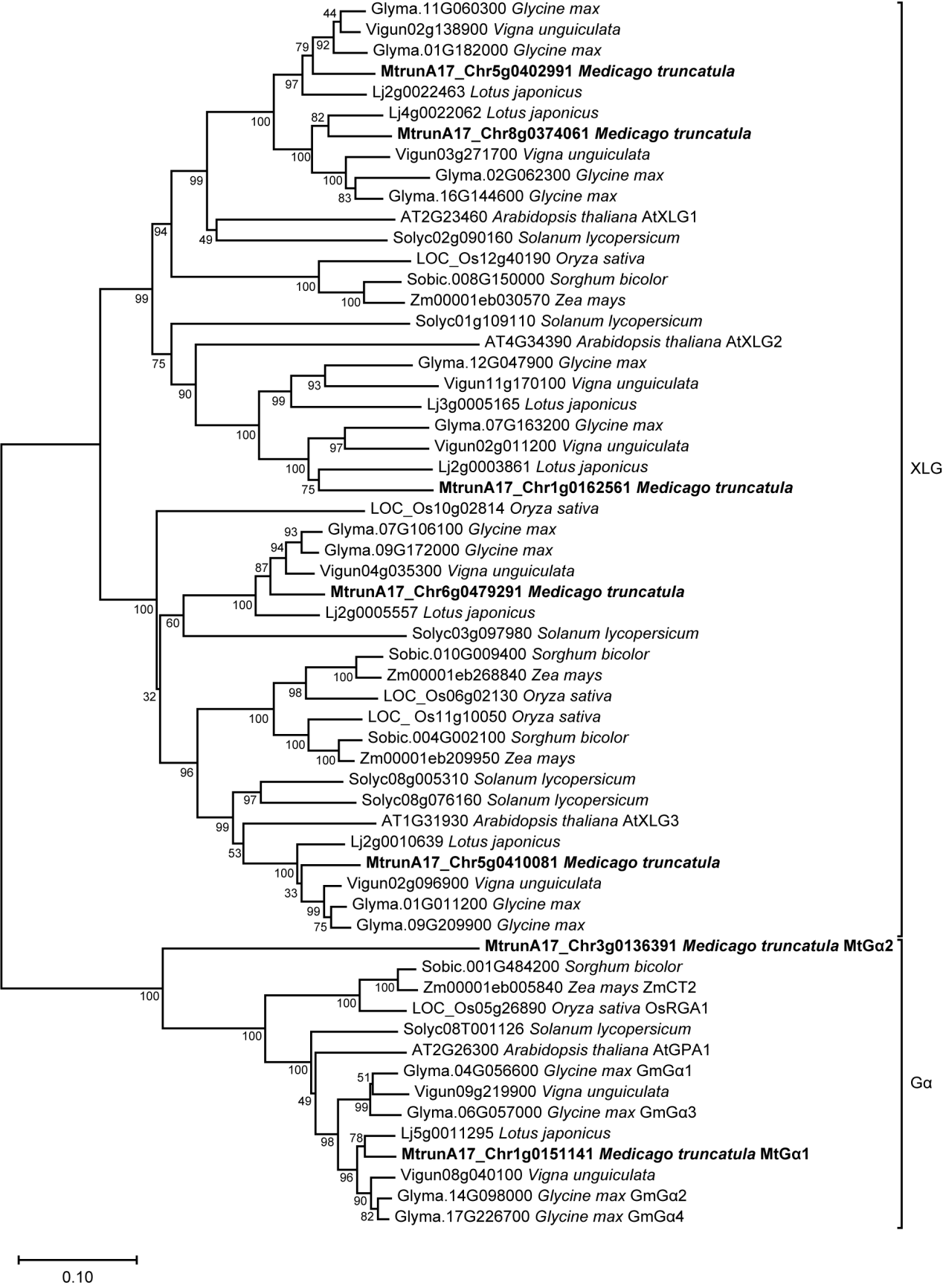


**Fig S1** Phylogenetic tree of Gα and XLG. Phylogenetic trees were constructed using the Neighbor-Joining (NJ) method based on protein sequences and displayed using MEGA11. The bootstrap value (1000 replicates) is shown next to the branches. The Gα and XLGs in *M.truncatula* are indicated in bold.


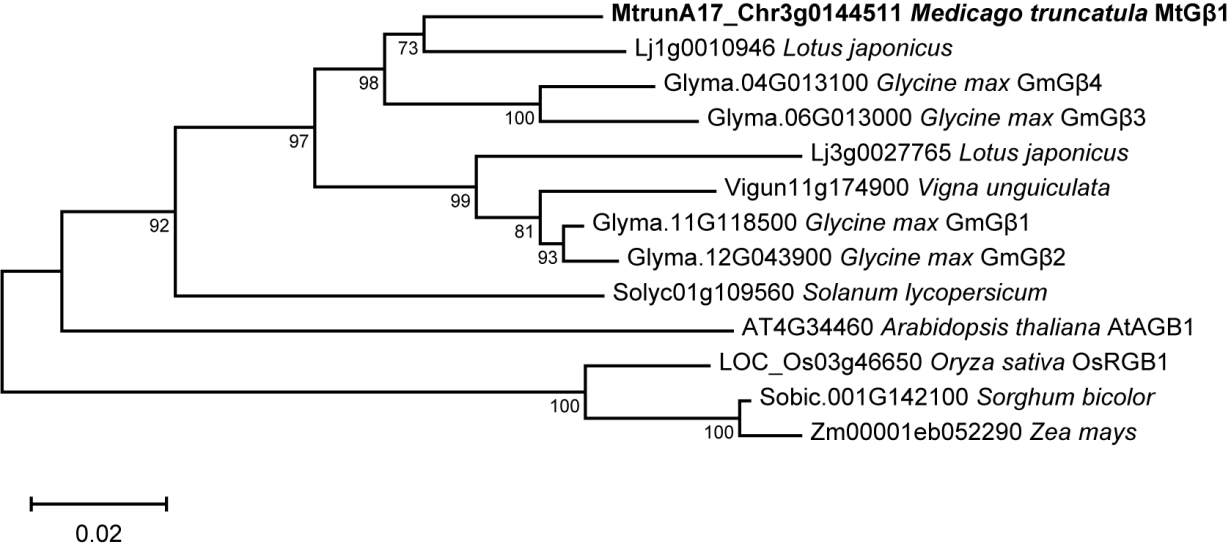


**Fig S2** Phylogenetic tree of Gβ. Phylogenetic trees were constructed using the Neighbor-Joining (NJ) method based on protein sequences and displayed using MEGA11. The bootstrap value (1000 replicates) is shown next to the branches. The Gβ in *M.truncatula* is indicated in bold.


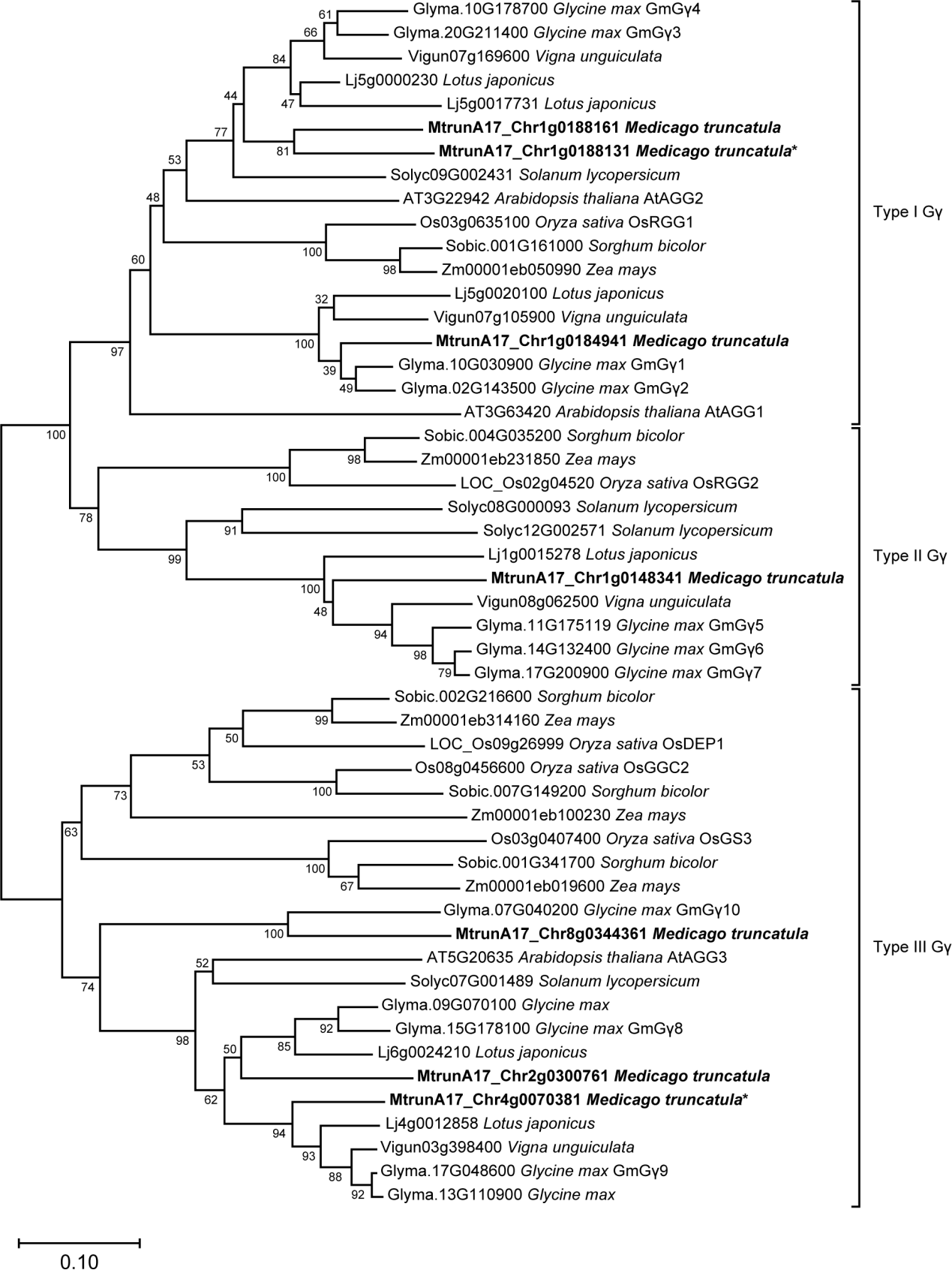


**Fig S3** Phylogenetic tree of Gγ. Phylogenetic trees were constructed using the Neighbor-Joining (NJ) method based on protein sequences and displayed using MEGA11. The bootstrap value (1000 replicates) is shown next to the branches. The Gγs in *M.truncatula* are indicated in bold. Asterisks indicate the new candidates of G protein components in *M.truncatula*.


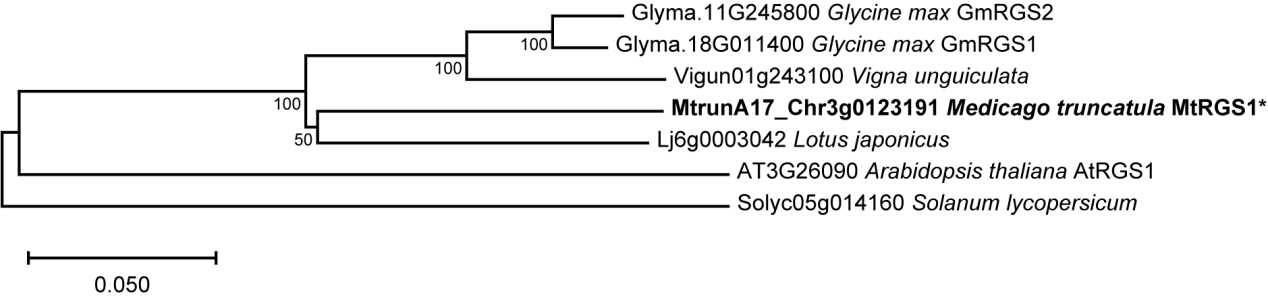


**Fig S4** Phylogenetic tree of RGS. Phylogenetic trees were constructed using the Neighbor-Joining (NJ) method based on protein sequences and displayed using MEGA11. The bootstrap value (1000 replicates) is shown next to the branches. The RGS in *M.truncatula* is indicated in bold. Asterisk indicates the new candidate of G protein components in *M.truncatula*.

**
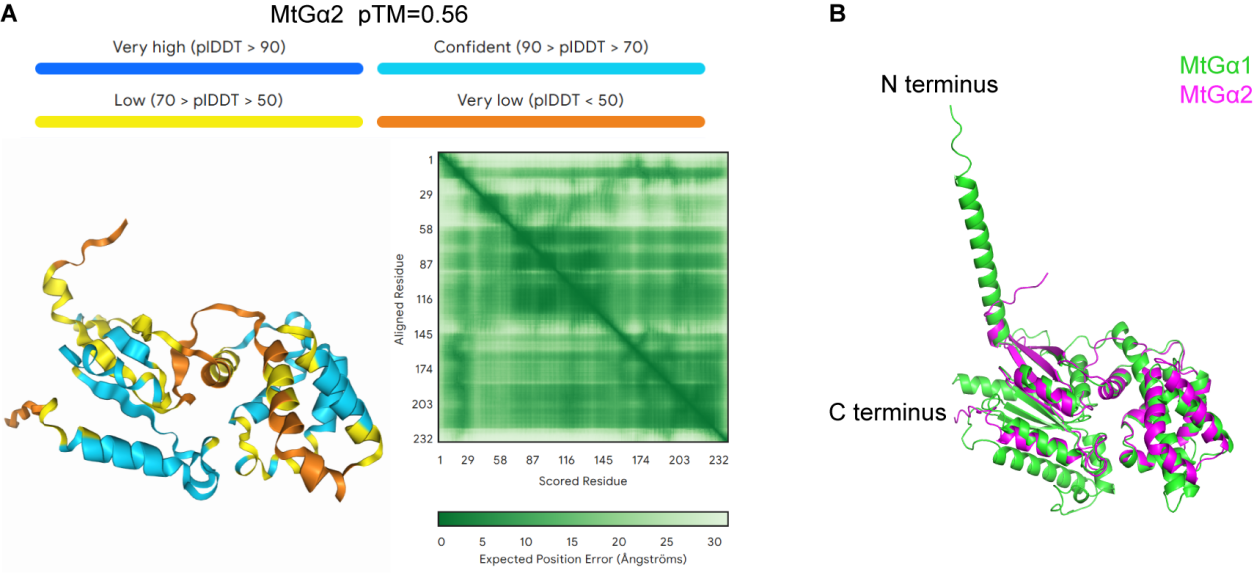
**

**Fig S5** AlphaFold3 model of MtGα2 protein. **A** The AlphaFold3 model of MtGα2 protein. **B** Superimposition of the predicted structures of MtGα1 (green) and MtGα2 (magenta).


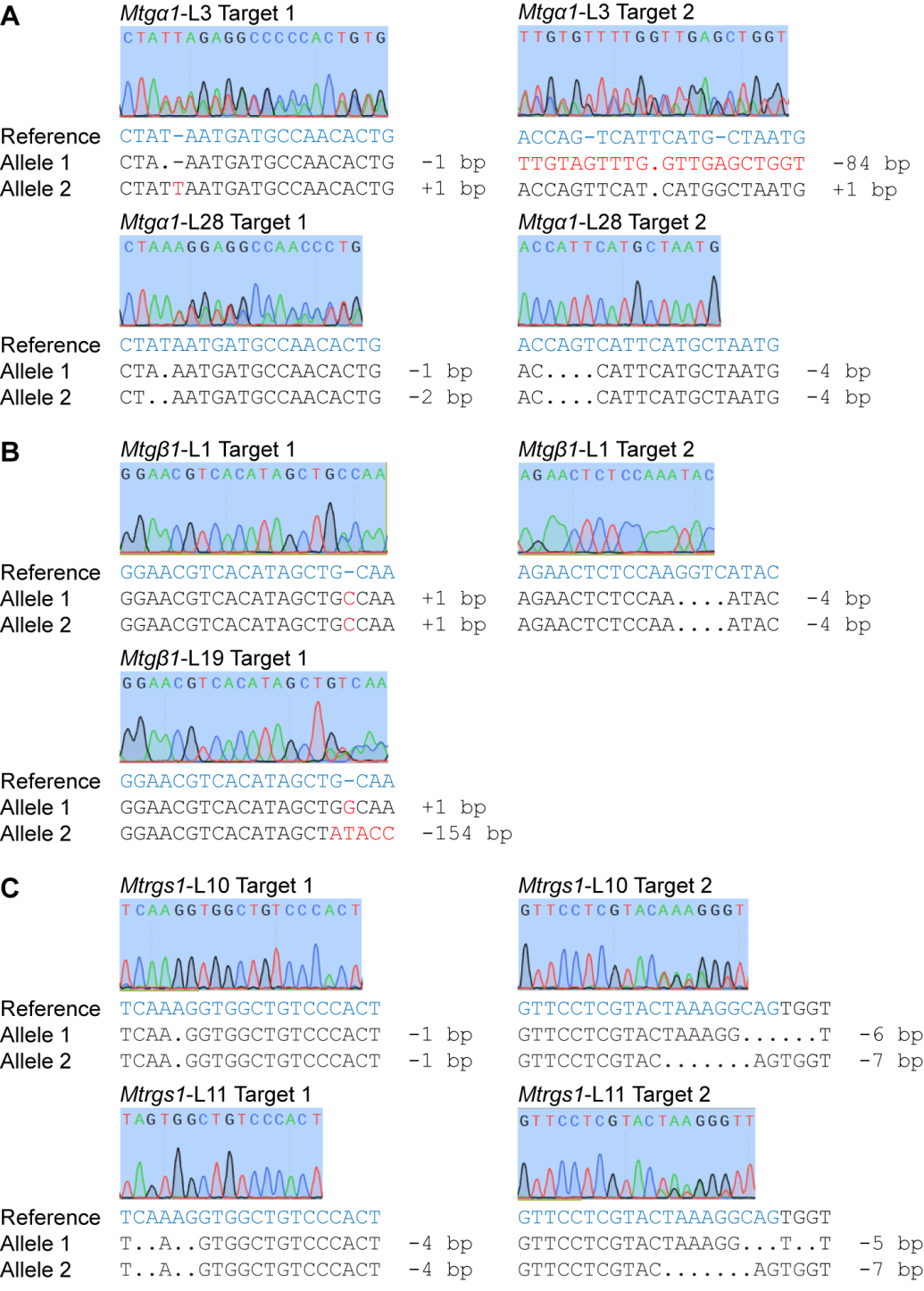


**Fig S6** Sanger sequencing of target sites for *MtGα1* (**A**), *MtGβ1* (**B**), and *MtRGS1* (**C**)*.* In the sequence analysis, sequences of the target sites are indicated in blue. Nucleotides in red represent insertion, and dots represent deletion. References represent the sequences of R108.


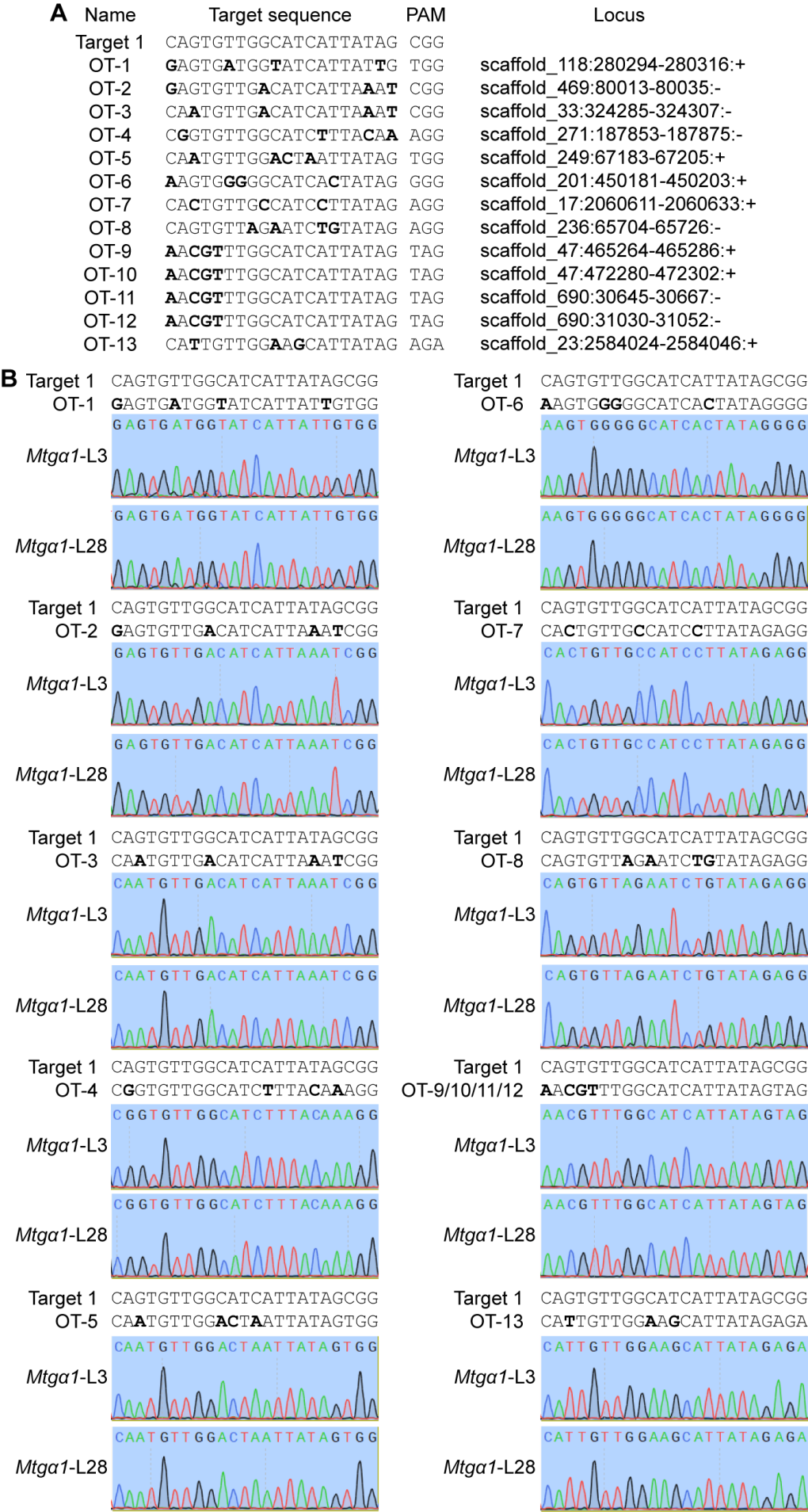


**Fig S7** Potential off-target sites for target 1 of the *MtGα1* sequence. **A** The potential off-target sites (OT) for target 1 of the *MtGα1* sequence. OT-1 to OT-13 represent 13 potential off-target sites. OT-9 to OT-12 show the same sequence with different loci. **B** Sanger sequencing of PCR amplicons that spanning the potential off-target sites. Mismatches to the target are indicated in bold.


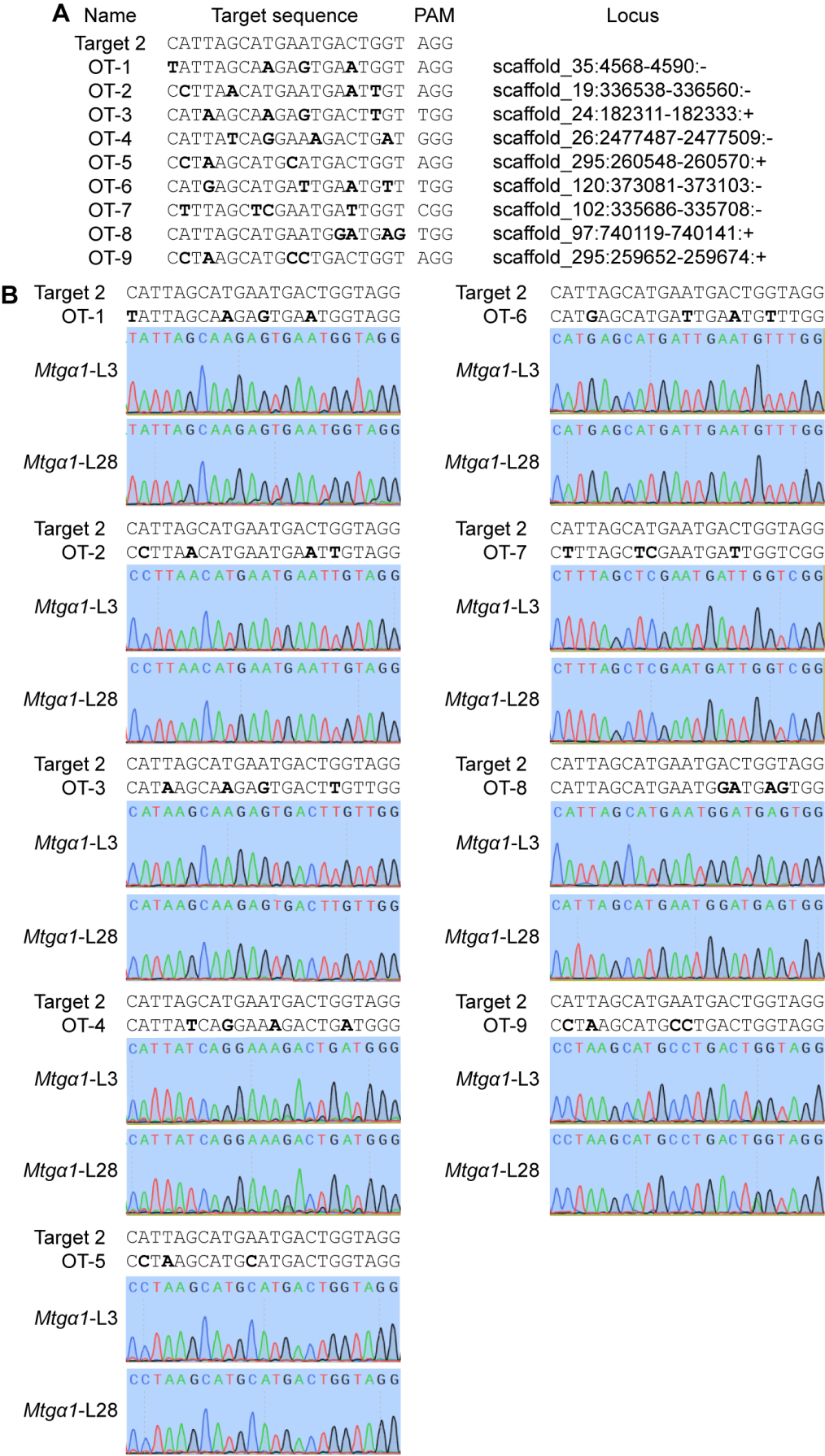


**Fig S8** Potential off-target sites for target 2 of the *MtGα1* sequence. **A** The potential off-target sites (OT) for target 2 of the *MtGα1* sequence. OT-1 to OT-9 represent 9 potential off-target sites. **B** Sanger sequencing of PCR amplicons that spanning the potential off-target sites. Mismatches to the target are indicated in bold.


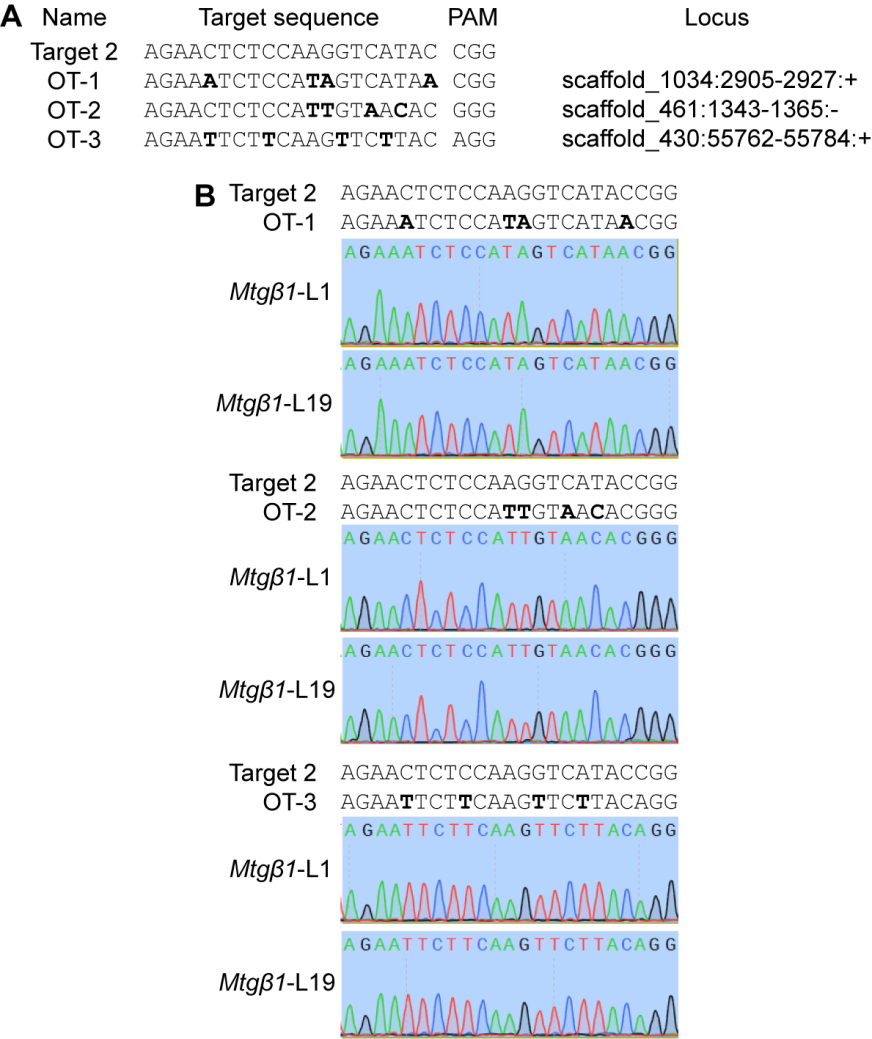


**Fig S9** Potential off-target sites for targets of the *MtGβ1* sequence. **A** The potential off-target sites (OT) for target 2 of the *MtGβ1* sequence. OT-1 to OT-3 represent 3 potential off-target sites. There have been no predicted potential off-target sites for target 1 of the *MtGβ1* sequence. **B** Sanger sequencing of PCR amplicons that spanning the potential off-target sites. Mismatches to the target are indicated in bold.


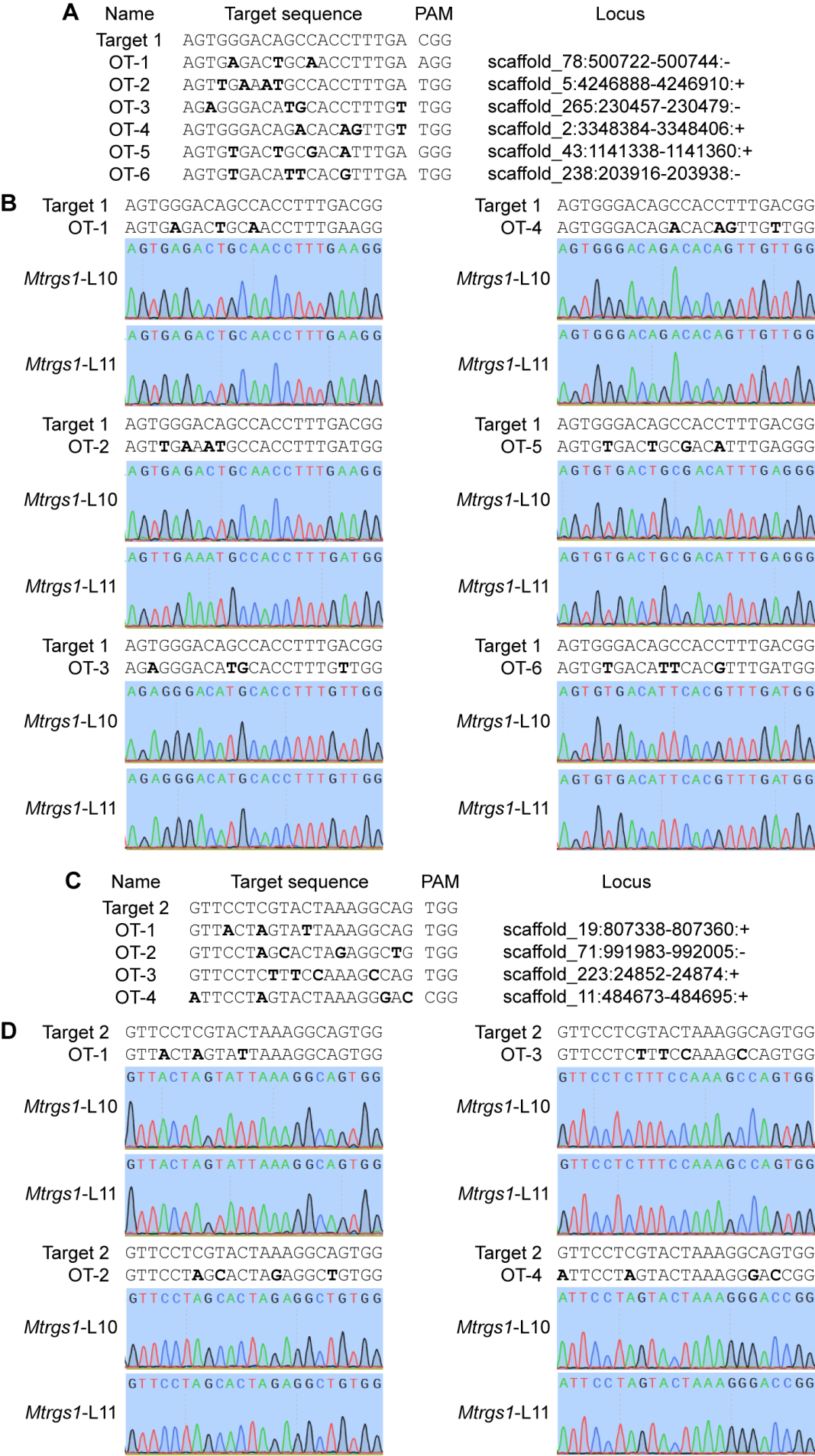


**Fig S10** Potential off-target sites for targets of the *MtRGS1* sequence. **A, C** The potential off-target sites (OT) for target 1 (**A**) and target 2 (**C**) of the *MtRGS1* sequence. OT-1 to OT-6 represent 6 potential off-target sites for target 1 (**A**), OT-1 to OT-4 represent 4 potential off-target sites for target 2 (**C**). **B, D** Sanger sequencing of PCR amplicons that spanning the potential off-target sites. Mismatches to the target are indicated in bold.


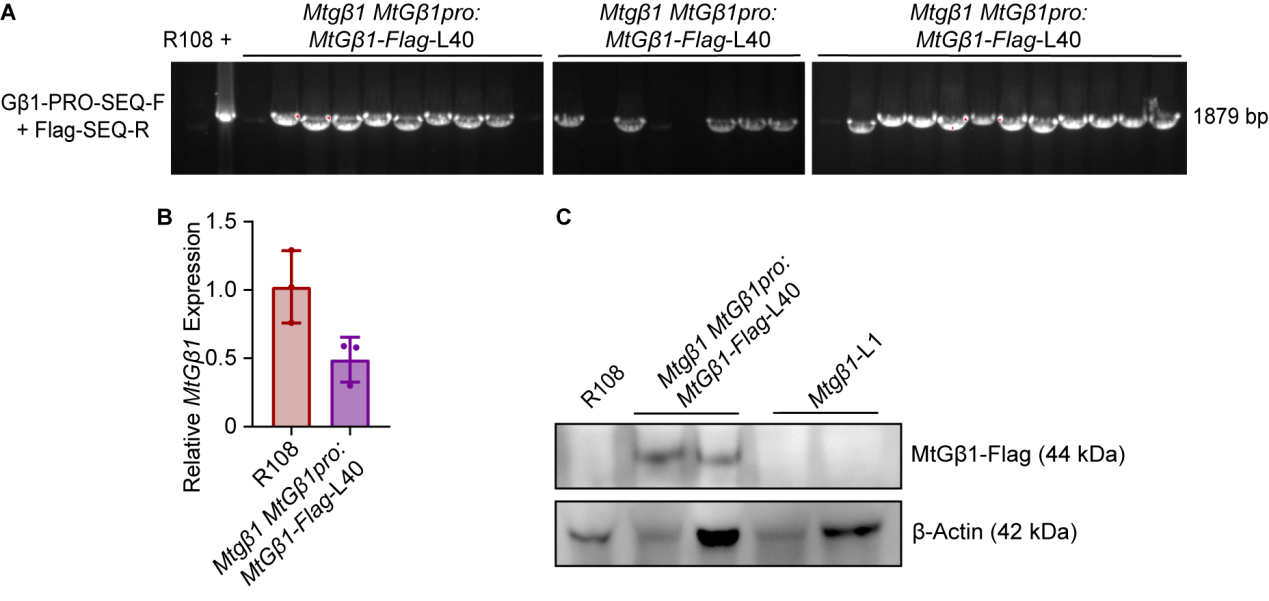


**Fig S11** The identification of *Mtgβ1 MtGβ1pro:MtGβ1-Flag*-L40 plants. **A** Identification of the T1 generation of *Mtgβ1 MtGβ1pro:MtGβ1-Flag*-L40 plants at the DNA level. The primers used in this assay are listed in supplementary table 1. **B** RT-qPCR analysis of the expression levels of *MtGβ1* in *Mtgβ1 MtGβ1pro:MtGβ1-Flag*-L40 and R108 leaves. The relative expression level of each gene was normalized to *MtACTIN4A*. Data represent means ± SD from three biological replicates. **C** Immunoblot analysis of *Mtgβ1 MtGβ1pro:MtGβ1-Flag*-L40, *Mtgβ1*-L1 and R108 plants. β-Actin protein was used as the loading control.


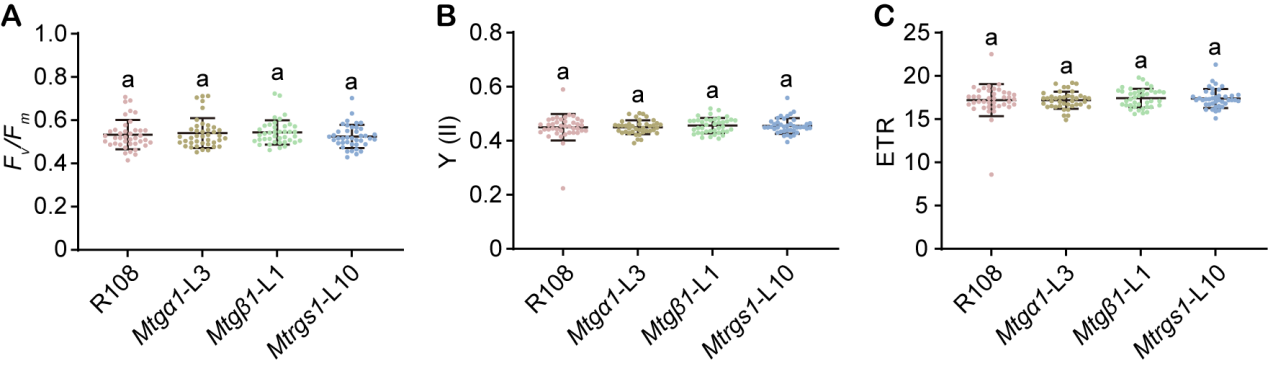


**Fig S12** Impact of *MtGα1*, *MtGβ1* and *MtRGS1* on the photosynthetic efficiency. **A–C** Quantification of maximum quantum yield of photosystem II (PSII) efficiency (*F_v_/F_m_*) (**A**), actual quantum yield (Ⅱ) [Y(Ⅱ)] (**B**), electron transport rate (ETR) (**C**). Germinated seedlings were grown in normal medium for 4 weeks. The horizontal lines represent the means (wider line) and the SD range; each dot represents an individual data point. Different lower-case letters indicate statistically significant differences, as determined by a one-way ANOVA followed by Tukey’s multiple comparison test. n = 40, *P* ＜ 0.05.

**Table S1 The list of primers used in this article**

| Primer Name | Sequence (5’-3’) | Usage |
| --- | --- | --- |
| MtGα1-RT-F | AGCTGGAGAGTCGGGGAAG | RT-qPCR |
| MtGα1-RT-R | GGATATAACATACTTCGAAAAATCAA | RT-qPCR |
| MtGβ1-RT-F | ACATGTGCTTTCTCCCC | RT-qPCR |
| MtGβ1-RT-R | TGAGTGTCTTCGCCTGGAAC | RT-qPCR |
| MtRGS1-RT-F | TTACCGTTGCCATTCTCTCC | RT-qPCR |
| MtRGS1-RT-R | TAGTGGACCTTCACCCCAAA | RT-qPCR |
| MtActin4A-RT-F | CCAAAGGCCAACAGAGAAAA | RT-qPCR |
| MtActin4A-RT-R | ACGACCAGCAAGATCCAAAC | RT-qPCR |
| MtActin11-RT-F | ACCCAAAGCATCAAATAATAAGTCAACC | RT-qPCR |
| MtActin11-RT-R | TGGCATCACTCAGTACCTTTCAACTG | RT-qPCR |
| MtRBP1-RT-F | AGGGGCAAGTTCCTTCATTT | RT-qPCR |
| MtRBP1-RT-R | GGTAGAAGTGCTGGCTCAGG | RT-qPCR |
| MtGα1-GFP-F | CTTGTCGACGGATCCATGGGCTTACTCTGTAGCAAAAG | Subcellular localization |
| MtGα1-GFP-R | ATCTACCATTCTAGATAACAAGCCAGCCTCAAAGAG | Subcellular localization |
| MtGβ1-GFP-F | CTTGTCGACGGATCCATGTCAGTTACGGAGCTGAAGG | Subcellular localization |
| MtGβ1-GFP-R | ATCTACCATTCTAGAAATCACCTTCCTATGCCCTCC | Subcellular localization |
| MtRGS1-GFP-F | CTTGTCGACGGATCCATGGCGAATTTCAAGTGTGCC | Subcellular localization |
| MtRGS1-GFP-R | ATCTACCATTCTAGATGAGTCGGTATCATTGCCGC | Subcellular localization |
| MtGβ1-NGFP-F | CGGAGGTGGCTCTAGAATGTCAGTTACGGAGCTGAAGG | Subcellular localization |
| MtGβ1-NGFP-R | TGGTCTTTGTAGTCACTAGTTCAAATCACCTTCCTATGCCCT | Subcellular localization |
| MtGα1-Target1-BsF | ATATATGGTCTCGATTGAGTGTTGGCATCATTATAGGTT | CRISPR-Cas9 |
| MtGα1-Target1-F0 | TGAGTGTTGGCATCATTATAGGTTTTAGAGCTAGAAATAGC | CRISPR-Cas9 |
| MtGα1-Target2-R0 | AACACCAGTCATTCATGCTAATCGTTAGATGGAGTGTGCTAGC | CRISPR-Cas9 |
| MtGα1-Target2-BsR | ATTATTGGTCTCGAAACACCAGTCATTCATGCTAATCGT | CRISPR-Cas9 |
| MtGβ1-Target1-BsF | ATATATGGTCTCGATTGGAACGTCACATAGCTGCAAGTT | CRISPR-Cas9 |
| MtGβ1-Target1-F0 | TGGAACGTCACATAGCTGCAAGTTTTAGAGCTAGAAATAGC | CRISPR-Cas9 |
| MtGβ1-Target2-R0 | AACGTATGACCTTGGAGAGTTCCGTTAGATGGAGTGTGCTAGC | CRISPR-Cas9 |
| MtGβ1-Target2-BsR | ATTATTGGTCTCGAAACGTATGACCTTGGAGAGTTCCGT | CRISPR-Cas9 |
| MtRGS1-Target1-BsF | ATATATGGTCTCGATTGGTGGGACAGCCACCTTTGAGTT | CRISPR-Cas9 |
| MtRGS1-Target1-F0 | TGGTGGGACAGCCACCTTTGAGTTTTAGAGCTAGAAATAGC | CRISPR-Cas9 |
| MtRGS1-Target2-R0 | AACCTGCCTTTAGTACGAGGAACGTTAGATGGAGTGTGCTAGC | CRISPR-Cas9 |
| MtRGS1-Target2-BsR | ATTATTGGTCTCGAAACCTGCCTTTAGTACGAGGAACGT | CRISPR-Cas9 |
| MtGβ1-PRO-1381-F | CGGCGCGCCGAATTCGCACTCTATGATGAA | Complementation test |
| MtGβ1-PRO-1381-R | CAGGTCGACGGATCCCTTTTTTAGGTCTTCCTT | Complementation test |
| pMtGβ1-MtGβ1-Flag-F | GACCTAAAAAAGGGATCCGTCGACATGTCAGTTACGGAGCTG | Complementation test |
| MtGβ1-Flag-R | GTAGTCACTAGTTCTAGAAATCACCTTCCTATGCCCTCC | Complementation test |
| pMtGβ1-MtGβ1-Flag-R | CCTCTTAAAGCTTGGCTGCAGGTCGACCTACTTATCGTCATCGTCC | Complementation test |
| MtGβ1-PRO-SEQ-F | ATGAAGTATGAAAGGATCAAGAGCAT | Sequencing for complementation test |
| Flag-SEQ-R | CATCGTCCTTGTAATCGATGTCGTG | Sequencing for complementation test |
